# Enabling high-throughput enzyme discovery and engineering with a low-cost, robot-assisted pipeline

**DOI:** 10.1038/s41598-024-64938-0

**Published:** 2024-06-24

**Authors:** Brenna Norton-Baker, Mackenzie C. R. Denton, Natasha P. Murphy, Benjamin Fram, Samuel Lim, Erika Erickson, Nicholas P. Gauthier, Gregg T. Beckham

**Affiliations:** 1 Renewable Resources and Enabling Sciences Center, National Renewable Energy Laboratory, Golden, CO USA; 2 BOTTLE Consortium, Golden, CO USA; 3 Agile BioFoundry, Emeryville, CA USA; 4 Department of Systems Biology, Harvard Medical School, Boston, MA USA; 5 Department of Data Sciences, Dana-Farber Cancer Institute, Boston, MA USA

**Keywords:** High-throughput screening, Isolation, separation and purification, Enzymes

## Abstract

As genomic databases expand and artificial intelligence tools advance, there is a growing demand for efficient characterization of large numbers of proteins. To this end, here we describe a generalizable pipeline for high-throughput protein purification using small-scale expression in *E. coli* and an affordable liquid-handling robot. This low-cost platform enables the purification of 96 proteins in parallel with minimal waste and is scalable for processing hundreds of proteins weekly per user. We demonstrate the performance of this method with the expression and purification of the leading poly(ethylene terephthalate) hydrolases reported in the literature. Replicate experiments demonstrated reproducibility and enzyme purity and yields (up to 400 µg) sufficient for comprehensive analyses of both thermostability and activity, generating a standardized benchmark dataset for comparing these plastic-degrading enzymes. The cost-effectiveness and ease of implementation of this platform render it broadly applicable to diverse protein characterization challenges in the biological sciences.

## Main

Exciting advancements in artificial intelligence and machine learning across nearly every industry, including the biological sciences, have demonstrated the power of big data, and the protein engineering field is poised to benefit immensely from this data revolution^1^. Innovations in computational methodologies have improved the prediction of protein properties from amino acid sequence and have empowered researchers to explore vast sequence spaces to identify proteins, and particularly enzymes, with desired properties^2–10^. Breakthroughs have propelled both the discovery of novel enzymes from natural diversity^11–15^ and the engineering of known enzymes to enhance properties such as activity, thermostability, pH optima, and solvent tolerance^16–20^. With many available techniques to discover and diversify sequences, the need to produce and analyze enzymes rapidly and efficiently has grown significantly.

Traditional approaches for laboratory enzyme production are commonly conducted using *Escherichia coli*-based expression at the liter-scale followed by chromatographic purification, but this conventional approach does not typically have sufficient throughput to meet the scalability needed to handle the increasing volume of candidate enzymes, especially in the era of machine learning. Studies that involve the evaluation of many enzyme variants often rely on cell lysate assays^21,22^, but typical analyses that evaluate biophysical characteristics, such as thermostability, cannot be performed without enriching sample purity. Additionally, activity assessments are most meaningful when the enzyme concentration is controlled, as lysates can vary significantly in expression level, not only between cultivations but also between similar enzymes. Cell-free expression offers a faster turnaround for protein production and potentially better tolerance of toxic proteins. However, yields are often low, and similar to standard recombinant expression, there is still a need to purify the target proteins for accurate biophysical and activity assessments^23^. Thus, there is a critical need for cost-effective, high-throughput purification and testing of enzymes.

Fortunately, this need arises in conjunction with the availability of liquid-handling robots that have enabled increased throughput with less human labor and reduced potential for error^24,25^. Considerable development in this area has been seen in the commercial sector with automation systems available at a wide range of prices. Liquid-handling systems, such as those provided today by Hamilton or Tecan are among the most flexible, but require significant user training and for many groups are prohibitively expensive with prices > $150,000 USD (at the time of writing) to access systems with the necessary capabilities. Studies showcasing high-throughput protein production and purification using these liquid handlers (or other similar commercial or custom systems) demonstrate the potential for automated approaches to expedite protein research, while also illustrating the financial investment and technical expertise required^26–29^. Other systems that are tailored specifically to biomolecule purification, such as the KingFisher, are well-suited to their application, but do not currently offer flexibility for use in other experiments. These systems are still expensive, with a price of ~ $80,000 USD (at the time of writing) for a unit that processes 96 samples. Several liquid-handling robotics platforms have emerged towards democratization of automation with lower prices, increased modularity, and easier protocol development. For example, the OT-2 from Opentrons costs ~ $20,000–30,000 USD (at the time of writing) for the robot equipped with pipettes and accessory modules. Further development and competition in the liquid-handler market are poised to even further reduce cost, increase ease of use, and promote accessibility of automation.

Development of a high-throughput expression and purification protocol integrated with a liquid-handling robot requires miniaturization of the process to align with well-plate formats for parallel processing. One significant advantage of miniaturization is the reduction of the material cost and experimental waste per sample. However, challenges emerge in the translation of large-scale techniques to small-scale, such as reaching adequate culture aeration, avoiding cross-well contamination, transferring low volumes without substantial sample loss, achieving sufficient final protein concentration for the desired assays, and ensuring compatible buffers for downstream analyses.

In this work, we aimed to address these challenges without the use of specialized equipment or expensive consumables and using open-source code to ensure the accessibility, affordability, and flexibility of the platform. Specifically, we present a protocol and accompanying robot-control scripts for the recombinant expression and purification of enzymes from *E. coli* assisted by a low-cost liquid-handling robot—here, Opentrons robot, OT-2, a low-cost liquid handler compatible with open-source protocols written in Python—and other common biochemical laboratory equipment. This platform, with accompanying easily adaptable Python code, enables the parallel transformation, inoculation, and purification of 96 enzymes in a well-plate format, with the option to process multiple plates consecutively, thus allowing hundreds of enzymes to be purified per week. As a proof-of-concept experiment, we demonstrate the expression, purification, and assay of a set of 23 poly(ethylene terephthalate) (PET) hydrolases sourced from the peer-reviewed literature, replicated across a 96-well plate. The semi-automated protocol produced purified samples with high reproducibility, both between wells and between trials. Sufficient yields and purity were achieved for both thermostability measurements and activity analysis on PET substrates across pH, temperature, and substrates. By examining many of the most-studied PET hydrolases to date in the same assay, we identified those with higher performance across multiple reaction parameters. Overall, the aim of this method is to increase the efficiency of high-throughput studies of enzymes, helping to accelerate the pace of investigation into enzymatic activities with implications for industrial and medical applications.

## Results

### Small-scale, robot-assisted protein expression and purification

**Figure 1:**
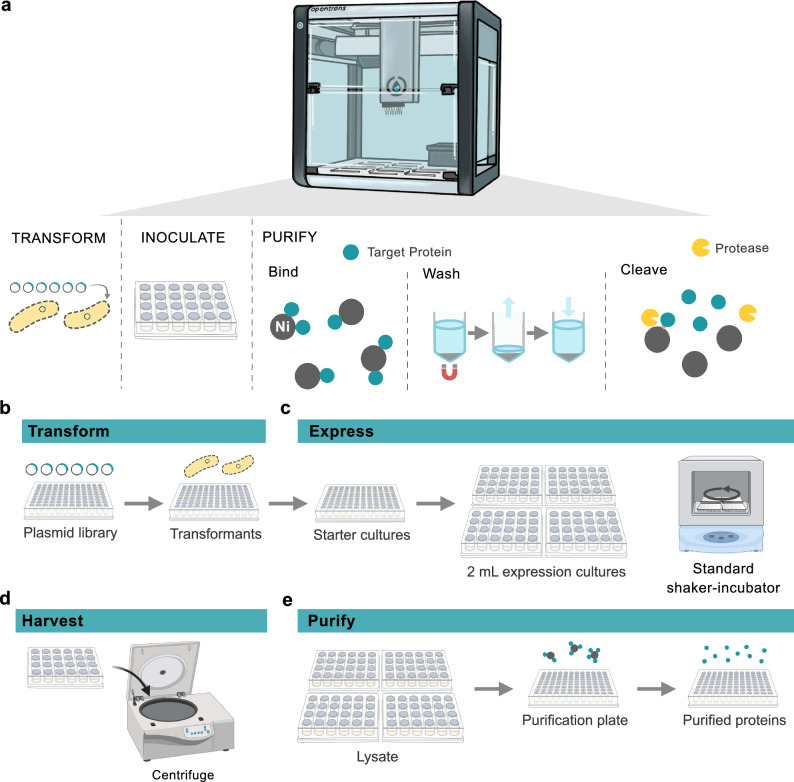
High-throughput expression and purification of proteins assisted by the OT-2. (**a**) Several key steps in the high-throughput expression and purification workflow that were automated using the OT-2: transformation of chemically competent *E. coli*, inoculation from 96-well starter cultures to four 24-well plates for expression, and Ni-charged magnetic bead purification of the recombinant proteins. The purification step involved binding the His-tagged target protein to the magnetic beads, washing away cell debris and non-target proteins, then cleaving the target protein off the magnetic beads with proteolytic cleavage. (**b**) The workflow began with transformation of a plasmid library into chemically competent *E. coli* cells, which were then grown to saturation. (**c**) The 96-well plate starter cultures were used to inoculate four 24-well plates containing 2 mL autoinduction media. The growth of the expression cultures occurred in a standard (19 mm orbit, 300 rpm) shaker-incubator until saturation. (**d**) The cells were then harvested via centrifugation. (**e**) The cells were resuspended in a chemical lysis solution and transferred via the OT-2 back to a 96-well plate for purification. The Ni-charged magnetic bead purification protocol, the most tedious step to perform without liquid handling assistance, employed the OT-2 magnetic module and performed washes to remove non-target proteins and add the protease for cleavage. A protease cleavage liberated the target proteins from the beads and the OT-2 performs a final transfer of the purified protein supernatant to a fresh plate for testing. Portions of this figure were created with BioRender.com.

To translate large-scale expression and purification methods to the liquid-handling robot, each step of enzyme production was miniaturized and adjusted for implementation in well-plates. The protocol design had several goals: automate the most tedious and human error-prone steps (Fig. 1a), enable rapid deployment of parameter changes (especially useful for testing), reduce the need for specialized equipment, minimize the use of consumables and waste generated, and achieve final purified protein in high yields and high purity. The protocols for each step are available (https://github.com/beckham-lab/opentrons) and more detailed instructions for their implementation are described in the Supplemental Information (SI). Supplementary Tables 1–3 detail the recommended equipment, possible equipment substitutions, labware selection, and buffer compositions.

#### Gene synthesis and cloning

We employed a plasmid construct containing both an affinity tag and a protease cleavage recognition site, specifically pCDB179, which confers a histidine tag for Ni-affinity purification and a SUMO site (Smt3) for proteolytic cleavage^30^. In the case of Ni-affinity, imidazole is typically used to elute the target protein, but its presence in high concentrations can interfere with subsequent analyses and would therefore require buffer exchange. Methods for buffer exchange of small volume samples in-plate remain limited, to our knowledge, with options proving tedious, dilutive, and costly. Instead, a protease cleavage served as the ‘elution’ step to release the target protein from the Ni-charged magnetic beads to avoid high final concentrations of elution agents. We chose SUMO as the fusion protein to allow for a scarless cleavage, thereby avoiding any effects from the chosen tag. The genes for the target proteins were codon optimized, synthesized, and cloned commercially, which proved to be the costliest step in this protocol. Other methods for gene synthesis or library generation to generate in-house sequences are beyond the scope of this study, but strategies to reduce the time and cost of construction of sequence libraries represent an active area of research^31–33^.

#### Transformation

To transform competent *E. coli* cells (Fig. 1b), we used a commercial kit (Zymo Mix & Go! *E. coli* Transformation Kit), which allows users to prepare competent cells that can be transformed by simple incubation of plasmid with competent *E. coli* without heat shock. This method substantially reduced cost compared to purchasing competent cells directly, improved reproducibility, and avoided human intervention in the transformation protocol. This choice also reduced waste by avoiding plate transfers, as heating each well evenly in a plate would involve the use of PCR plates, while growth is better performed in deep-well plates. Translation of this step into the robot required the use of a cooling block, a module available for purchase as an add-on to the OT-2. However, this step is also easily performed by hand via a multichannel pipette, such that this module is not required for use of this protocol.

For transformation, the chemically competent *E. coli* cells were combined with plasmid then incubated on ice. An outgrowth step was performed, then antibiotic was added and growth continued to saturation. By growing the transformation mix directly for use as starter cultures to inoculate expression cultures, this method bypasses the need for plating transformations and picking colonies. This approach offers substantial time and cost savings for a step that is difficult to automate and literature supports that this method does not impair recombinant protein production^34,35^. Growth for ~ 40 h at 30 °C was found to yield sufficiently saturated cultures for inoculation into the expression media. Shorter growth periods at 30 and 37 °C were tested but it was found that the cultures did not reach saturation by just a single overnight step. Consequently, a lower temperature and longer growth time were favored. Two transformation protocols are supplied in the available code (Supplementary Table 4). The first is a plate-to-plate transformation that uses a multichannel pipette to transfer all wells from a source plasmid plate to the destination plate containing competent cells. The second is a ‘cherry-picking’ transformation that allows the user to specify up to 3 source plates of plasmids and the desired destination wells for each to build custom expression plates from multiple plasmid libraries.

#### Inoculation

In the next step, expression media was inoculated (Fig. 1c). Autoinduction was chosen to further reduce human intervention as it avoids the need to monitor cell density to determine time of induction. To improve aeration and increase culture volume for higher yields, 24-deep-well plates were used with 2 mL cultures. The 24-well plates with 10 mL well volumes also supported the use of standard shaker-incubators with larger orbits (here, 19 mm) rather than specialized plate shaker-incubators with smaller orbits, typically 3 mm. We performed some preliminary investigations into using 96-deep-well plates for expression to avoid the need for plate reformatting. Generally, we observed with 0.5 mL cultures in 2 mL wells in 96-well plates did not sustain the mixing necessary for sufficient aeration of autoinduction cultures to reach high cell densities. IPTG-induction may offer a preferred route if growth in 96-well plates is desired. We observed that even high expressing proteins in 24 wells plates often did not exceed quantification thresholds when grown in 96-well plates. The robot was used to inoculate from the 96-well plates used in transformation to four 24-well plates used for expression. Three inoculation protocols are available for this step (Supplementary Table 5). The first method uses a single channel pipette to transfer the inoculum to the expression plate individually for each well. To decrease protocol time, an alternative method was developed that uses a half-loaded 8-channel pipette to inoculate 4 wells simultaneously. This method resulted in rows A, C, E, and G being grouped on to the first two 24-well plates and B, D, F, and H being grouped on the next two 24-well plates. This ‘row-swap’ method was later reversed upon return to a 96-well plate for purification, but users should be cautious to keep note of well identity if this method is used. The time required for inoculation was reduced from approximately 1 h to 15 min using the ‘row-swap’ method, and additional time was saved in the transfer back to a 96-well plate for purification, also reducing this step from approximately 1 h to 15 min. The final method is a ‘cherry-picking’ inoculation that allows the user to specify up to 3 source plates of starter cultures to use to build custom expression plates.

#### Expression

After the four 24-well plates were inoculated, the plates were sealed with gas permeable seals and grown to saturation. The expression protocol was growth at 37 °C for several hours to reach OD_600_ ~ 1 then 18 °C for ~ 40 h. This low temperature expression protocol was chosen to support high expression levels for lower stability proteins and still provided high expression levels for high stability proteins, allowing a more generalizable protocol. This step may be optimized towards the proteins studied and we have also found 25 °C for 24 h to be effective for the enzymes studied here. An expression reporter, red fluorescent protein (RFP), was used as a visual indicator of recombinant protein levels. After expression was complete, the RFP-containing wells exhibited a bright pink color (Supplementary Fig. 1). Cells were sedimented by centrifugation and the supernatant was discarded (Fig. 1d).

#### Lysis

The purification (Fig. 1e) began with cell resuspension in lysis buffer; here, we performed a chemical lysis using a detergent (*n*-octyl-β-D-glucopyranoside) and supplementation of the lysis buffer with DNaseI and lysozyme. Cell resuspension and lysis proceeded for 1 h, then Ni-charged magnetic beads were added without clarifying the lysate. By avoiding clarification, this protocol further reduced human intervention and the use of additional plates and pipette tips. Binding of the His-tagged target proteins proceeded for 2 h. The lysate was aspirated (Supplementary Table 6) and the magnetic beads were resuspended in fresh buffer. To aid in this aspiration step, we include in the SI an assembly of a home-built magnetic module plate using bar magnets and a deep-well 96-well plate that costs under $10 in parts to assemble, allowing all four 24-well plates to be aspirated in a single protocol without requiring four OT-2 magnetic modules (Supplementary Fig. 2). The magnetic bead suspensions in four 24-well plates were then transferred to one 96-well plate for a faster purification with a multichannel pipette on the robot. Two protocols are available for this transfer (Supplementary Table 7). Like before, one method transfers the contents of each well back to its corresponding well in the 96-well plate via a single channel pipette, whereas the second option uses a half-loaded multichannel pipette to reverse the ‘row-swap’ inoculation, if performed, to restore the original plate map and reduce the time of this transfer step.

#### Purification

Next, the robot was used to perform the most pipetting-intensive step, namely the washing of the magnetic beads to remove cell debris and non-target proteins. Two purification protocols are available for this step (Supplementary Table 8), both of which use the OT-2 magnetic module. The first method also uses the heater-shaker module to perform the mag-bead mixing steps. As the OT-2 cannot move plates, this requires the user to move the plate between the magnetic module and the shaker module. The second method uses only the magnetic module, and the pipette is used to mix the mag-beads for wash steps, thus avoiding user intervention. This ‘pipetting-only’ method requires an increased number of wash steps, but both methods take approximately the same amount of time. The washes consisted of first a low concentration imidazole-containing buffer to remove non-specific bound proteins, then the desired final buffer that does not contain imidazole. The final buffer may be tailored to the end use for the experiment but it must be compatible with protease function. Typical conditions for SUMO protease cleavage range from pH 7–9 with 100–300 mM NaCl at 4–30 °C, but the enzyme displays activity under a wide range of conditions even in the presence of various additives^36^. After the wash steps, protease was added to each well to initiate the cleavage of the target protein off of the mag-beads. After cleavage was complete (~ 3–4 h at room temperature), the robot performed the transfer of the supernatant off the mag-beads to a new plate.

### Assessment of expression yield and thermostability

**Figure 2:**
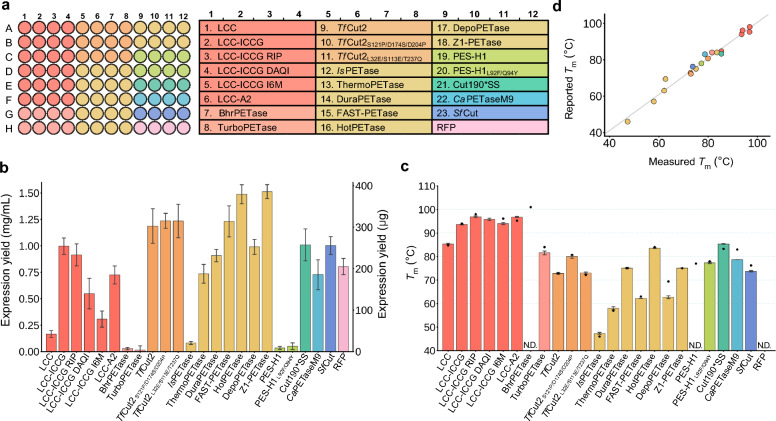
Expression yields and thermostability. (**a**) The plate map for expression and purification with each variant replicated in four adjacent wells. Color groupings indicate a naturally occurring enzyme (first in each grouping if present) and its engineered variants. For Cut190*SS and *Ca*PETaseM9, only engineered variants were studied. Red fluorescent protein (RFP) was also included as a visual reporter of expression levels. (**b**) Expression yields from a single well shown in mg/mL concentration on the left and total µg on the right for each PET-hydrolase as well as RFP from three separate rounds of expression and purification with four replicate wells in each round. Data are presented as mean values ± standard deviation (*n* = 12). (**c**) Thermostability measurements for each protein measured with differential scanning fluorimetry (DSF) using Sypro Orange dye. Data are presented as mean values ± standard deviation (*n* = 4). Black dots represent the literature-reported *T*_m_ value for each enzyme. N.D. = not determined. For BhrPETase, its reported *T*_m_ of 101 °C was above the temperature limit of the instrument used here. For RFP, its visible-range absorbance likely interfered with the dye fluorescence read. (**d**) A comparison of the reported *T*_m_ values for each enzyme with the *T*_m_ values measured in this study. Source data for this figure are provided in the Supplementary Source Data file.

A set of 23 PET hydrolases, all previously reported, was chosen to demonstrate the utility of this method, as they are a well-studied class of enzymes with potential to improve plastic waste recycling. Leaf-branch compost cutinase (LCC) was first reported in 2012 and its activity and thermostability improved via four mutations yielding LCC-ICCG^37,38^. Further engineering of LCC-ICCG led to the development of LCC-ICCG RIP, LCC-ICCG DAQI, LCC-ICCG I6M, and LCC-A2^39–42^. BhrPETase comes from the bacterium HR29 and an engineered variant, TurboPETase, was reported with increased activity^43,44^. *Tf*Cut2, a cutinase from *Thermobifida fusca,* has been engineered to produce improved variants *Tf*Cut2_S121P/D174S/D204P_ and *Tf*Cut2_L32E/S113E/T237Q_^45–47^. *Is*PETase was identified from *Ideonella sakaiensis*, a bacterium capable of using PET as its major carbon source, and has been engineered extensively, with ThermoPETase, DuraPETase, FAST-PETase, HotPETase, DepoPETase, and Z1-PETase among the notable variants^21,48–52^. PES-H1 (also known as PHL-7), isolated from compost metagenomic data, was engineered to yield a more active variant, PES-H1_L92F/Q94Y_^53,54^. The engineered variants Cut190*SS and *Ca*PETaseM9 were used in this study without their corresponding naturally-occurring parent protein^55,56^. The naturally-occurring enzyme *Sf*Cut from *Saccharopolyspora flava* was identified from a machine-learning guided natural diversity search for thermostable PET hydrolases^12^. Taken together, these enzymes were selected to compare activity of engineered variants against parent enzymes and to capture a range of various PET-depolymerizing enzymes from different source organisms and with diverse thermostability and activity profiles (Fig. 2a).

Concentration of purified proteins was assessed using the bicinchoninic acid (BCA) assay, providing a colorimetric readout of protein concentration based on binding with a copper-containing reagent. An OT-2 protocol was developed to perform the BCA assay by transferring sample from each well and controls of bovine serum albumin (BSA) at a known concentration, then adding the BCA reagent (Supplementary Table 9). Reproducibility of the expression and purification was demonstrated with four replicates in the same plate in three separate rounds of expression (Supplementary Fig. 3). We hypothesize that variation in expression yields from culture to culture may be influenced by differences in the cell density of the inoculation volume and its impact on the timing of expression. Saturating the starter culture helps to mitigate this effect, although some proteins may still be sensitive to variations in subsequent induction timing. Figure 2b shows the average expression yields for all trials. Out of the 24 proteins studied (23 PET-hydrolases and RFP), 19 expressed at levels above the threshold of definitive detection in the BCA assay (0.1 mg/mL). The five enzymes that failed to express sufficiently for quantification were *Is*PETase, BhrPETase and its variant TurboPETase, and PES-H1 and its variant PES-H1_L92F/Q94Y_. Enzyme purity and identity was confirmed with SDS-PAGE and, for select samples, intact mass spectrometry (Supplementary Figs. 4–17). SDS-PAGE analysis was additionally performed on multiple stages of the purification for those enzymes with insufficient yields to determine if failure occurred due to lack of expression, insolubility, poor binding to the Ni-charged beads, or insufficient cleavage of the fusion protein (Supplementary Fig. 18). The results suggest that low expression was a major factor in insufficient yield with no strong overexpression bands observed. Some samples also showed a fraction of incomplete cleavage of the fusion protein as evidenced in the final imidazole elution from the magnetic beads. For troubleshooting this protocol, especially when studying large sets of similar enzymes, we recommend performing this type of purification analysis on a smaller test set before committing to the construction of a library in a particular construct or strain. While the SUMO tag has been used successfully on a variety of proteins^36,57,58^, this protocol would be amenable to other constructs that confer an affinity tag^59^ and protease cleavage site^60^ and different *E. coli* strains can also influence expression efficiency^61^.

We next normalized the purified protein to set concentrations for downstream analyses. A script was developed and is available with the accompanying code to input the BCA concentration data and output an OT-2 protocol to dilute each well with buffer to the desired concentrations (Supplementary Table 10). Due to a broad range of starting concentrations for different enzymes, each well was diluted to either 0.1 or 0.3 mg/mL, as some wells contained high enough concentrations that normalization to 0.1 mg/mL would have exceeded the 2 mL volume of the well. For wells with concentrations < 0.1 mg/mL, no concentration adjustment was performed.

Thermostability was then measured using differential scanning fluorimetry (DSF) with Sypro Orange dye. In this assay, the hydrophobic binding dye increases in fluorescence as the protein unfolds and exposes hydrophobic regions. The point at which half of the protein is unfolded is the characteristic melting temperature, *T*_*m*_, also called the midpoint of unfolding. An OT-2 protocol was developed to facilitate the setup of the PCR plate for DSF (Supplementary Table 11), which was performed on a real-time PCR instrument. The measured values for the *T*_m_ of the studied enzymes are shown in Fig. 2c, along with the *T*_m_ values previously reported for each enzyme. To our knowledge, a *T*_m_ value has not been reported for LCC-ICCG DAQI. The measured *T*_m_ from DSF correlated well with the reported *T*_m_ values for all enzymes with reported values (Fig. 2d). For TurboPETase and PES-H1_L92F/Q94Y_, despite low enzyme yields after purification, *T*_m_ values were measured that corresponded with the reported *T*_m_ for each enzyme. The *T*_m_ values were not determined for 3 proteins: (1) for BhrPETase, the reported value for its *T*_m_ was 101 °C, which is above the temperature limit of the instrument used in this study, (2) no *T*_m_ was determined for PES-H1, most likely due to insufficient protein concentration, and (3) for RFP, its own excitation/emission properties in the visible range likely interfered with the dye based DSF measurement^62^. For all samples for which a *T*_m_ was successfully obtained, three showed evidence of two inflection points (Z1-PETase, DuraPETase, and DepoPETase.) *Is*PETase showed very low intensity, possibly obscuring multiple inflection points. Overall, most samples showed a single inflection point, further supporting high purity (Supplementary Fig. 19).

#### Comparison of PET hydrolysis activity

The successfully expressed and purified PET hydrolases were then selected for activity assessment based on their high expression yields and *T*_m_ values above 70 °C, resulting in 14 enzymes selected for assessment of their PET-hydrolase activity at elevated temperatures. To facilitate rapid addition of enzymes to assay plates via a multichannel pipette, the enzyme wells were pooled when necessary, normalized to 0.1 mg/mL, and reordered in two columns of a fresh plate. To aid reproducibility, two commercially available PET substrates were tested, both an amorphous film and a crystalline powder, with reported crystallinities at 4.0 ± 2.0%, and 39.3 ± 2.0%^12,63^, respectively. The pH conditions included 4.5 (NaCitrate), 5.5 (NaCitrate), 6.5 (NaPhosphate), 7.5 (NaPhosphate), 8.5 (glycine), and 9.5 (glycine). No additional salt was included in these assays. The two temperatures chosen for the study were 60 and 70 °C. The assay plates containing substrate and buffer were preheated to the reaction temperature with a pre-incubation of 2 h. The enzymes were added, then the plate was sealed and incubated at temperature with shaking. Two timepoints were measured on separate reaction plates with ultraviolet–visible (UV–Vis) spectroscopy data collected at 2 and 24 h, and high-performance liquid chromatography (HPLC) analysis also collected for the 24 h time point^12,64^. In total, each enzyme was tested in 48 conditions (6 different pH values, 2 substrates, 2 temperatures, 2 timepoints) in duplicate, totaling 96 reactions per enzyme. To each reaction, 2.5 µg of enzyme was added, requiring a minimum of 240 µg each enzyme for assaying under all conditions. However, it is worth noting that most preliminary screenings for activity would not require this amount of enzyme.

**Figure 3:**
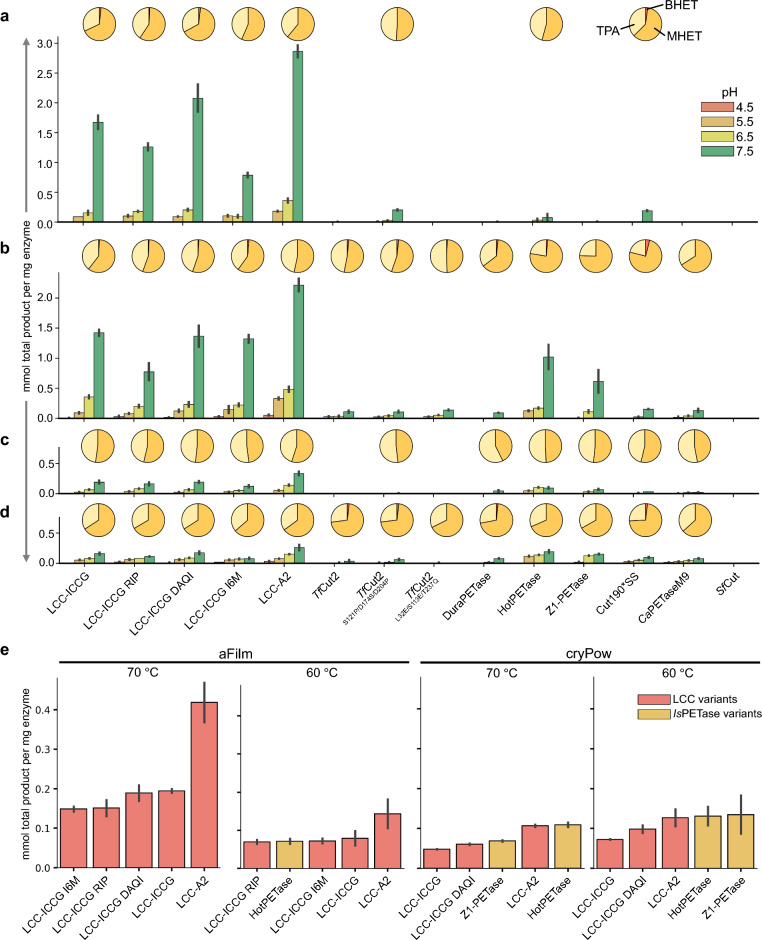
PET-hydrolysis activity. Enzymes with sufficient expression yields and *T*_m_ values greater than 70 °C were assayed for PET-hydrolysis activity for 6 pH values (4 displayed here), 2 temperatures, 2 substrates, 2 timepoints, in duplicate. The results are shown for (**a**) amorphous film (aFilm) at 70 °C, (**b**) aFilm at 60 °C, (**c**) crystalline powder (cryPow) at 70 °C, and (**d**) cryPow at 60 °C with bar charts showing mmol product formation per mg of enzyme added after 24 h for pH 4.5, 5.5, 6.5, and 7.5 as determined by UV–Vis analysis. The error bars represent the range between two biological replicates. The pie charts above each pH series show the product ratios of TPA, MHET, and BHET after 24 h for pH 7.5 determined by HPLC analysis. (**e**) Product formation after 2 h for the five top-performing enzymes in each condition at pH 7.5 determined by UV–Vis analysis. From *left* to *right*: aFilm at 70 °C, aFilm at 60 °C, cryPow at 70 °C, and cryPow at 60 °C. The error bars represent the range between two biological replicates. Source data for this figure is provided in the Supplementary Source Data file.

Select data are shown in Fig. 3 for the performance of each enzyme on amorphous film at 60 (Fig. 3a) and 70 °C (Fig. 3b) and crystalline powder at 60 (Fig. 3c) and 70 °C (Fig. 3d) at pH 4.5, 5.5, 6.5, and 7.5. Reactions at pH 8.5 and 9.5 used glycine buffer, which proved to be unfavorable for these assays, with sharp decreases in activity at 8.5 and recovery of some activity at 9.5 (Supplementary Fig. 20). The bar charts depict the normalized product quantities in millimoles (mmol) of total product equivalence after 24 h, determined through UV–Vis analysis of the liberated aromatics: terephthalic acid (TPA), mono(2-hydroxyethyl) terephthalate (MHET), and bis(2-hydroxyethyl) terephthalate (BHET). For all enzymes with activity, the peak performance was observed at pH 7.5, with a steep decline in activity corresponding with decreasing pH. *Sf*Cut showed no activity in any condition in the assay conducted here, possibly due to the high temperatures used or the low ionic strength of these reactions; previous results showed very low activity at these temperatures^12^. For all substrates and temperatures at pH 7.5, LCC-A2 was the highest-performing enzyme. The pie charts above each pH series indicate the product ratios from the 24 h timepoint of pH 7.5 determined by HPLC. MHET was the major product in most reactions and BHET was either a very minor product or below detection limits. Supplementary Fig. 21 demonstrates agreement between UV–Vis and HPLC analyses in determining total aromatic product.

The 2 h timepoint also allowed for the assessment of initial enzyme activity in each condition. Select data for pH 7.5 are shown in Fig. 3e, with the additional data for all of the reactions at 2 h in Supplementary Fig. 22. The normalized product (mmol per mg enzyme) from two replicates was determined by UV–Vis analysis and the top five performing enzymes for each substrate and temperature tested are shown. LCC-A2 demonstrated the highest activity on amorphous film (aFilm) at 70 and 60 °C. For the highest performers on crystalline powder (cryPow), LCC-A2 and HotPETase showed comparable activities at 70 °C and LCC-A2, HotPETase, and Z1-PETase show comparable activities at 60 °C.

## Discussion

Progress in protein discovery and design has accelerated the ability to identify desirable protein sequences, but many laboratories face limitations in analyzing these proteins at a pace that aligns with the capacity to generate candidate sequences. Lowering the cost and increasing the automation of this process are critical to increase throughput. Therefore, we aimed to develop a protocol using the OT-2 to provide a low-cost option for the purification and analysis of enzymes, or other proteins, making high-throughput studies more accessible to a broader range of research laboratories. Beyond increasing efficiency, this automation-assisted approach reduces the labor burden on researchers and lowers the risk of repetitive use injuries.

The full protocol is a five-day process from transformation to obtaining purified protein. This duration includes two days for the saturation of transformation cultures, followed by an additional two days for the low-temperature expression cultures to reach saturation. These transformation and expression steps involve less than 1 h of set-up with little human intervention otherwise. The lysis and purification can be completed in one day, with ~ 5 short interactions of the user with the robot to setup and initiate each step in the protocol. The protocol allows for the efficient purification of a full plate of 96 enzymes simultaneously and can be readily scaled for a single operator with a single robot to process multiple plates in a continuous workflow, enabling the purification of hundreds of enzymes weekly.

To demonstrate the performance of this protocol, we expressed and purified a set of PET-depolymerizing enzymes reported in the literature. Replicate wells and replicate trials showed the expression and purification yield to be reproducible. Our findings indicated high yields with the average yield of approximately 200 µg in these small-scale cultures being equivalent to 100 mg/L of culture. SDS-PAGE and intact mass spectrometry confirmed sample purity and identity, demonstrating low amounts of impurities and no evidence of cross-well contamination. Five enzymes failed to reach expression yields above the detection threshold, with potentially influencing factors such as codon usage, construct design, cell strain selection, and conditions during expression and purification. Screening through these conditions is another opportunity where this high-throughput platform could be applied via the small-scale and automated testing of constructs, strains, and conditions to optimize expression efficiency. Recently, an automated protocol utilizing an OT-2 was reported for testing various vectors and *E. coli* expression strains^61^. Employing this strategy to optimize expression efficiency, followed by high-throughput purification as described in this manuscript, may help reduce protein production as a bottleneck in enzyme engineering.

Expression yield and sample purity were sufficient to determine thermostability for almost all enzymes. Even for samples with low concentrations (< 0.1 mg/mL), *T*_m_ values were obtained that align with previously reported values and low concentration likely accounted for only one failed *T*_m_ measurement. Evaluation of thermostability underscores the benefits of high-throughput purification, where biophysical data can be obtained on isolated samples. Thermostability is a critical factor in many applications, especially industrially, where maintaining activity at high temperatures can be essential. Recent innovations in characterization instruments have substantially lowered sample volume requirements, allowing the measurement of other biophysical characteristics such as thermal aggregation propensity (*T*_agg_), isothermal stability, protein–protein interaction (k_D_, B_22_, and G_22_), and more, making many other analyses possible with the yields generated by this protocol^65,66^.

Activity testing also demonstrated the power of this platform, with yields sufficient for the testing of enzymatic activity in a concentration-normalized assay across a broad range of conditions, including 6 pHs, 2 temperatures, 2 substrates with 2 timepoints, all in duplicate (i.e., 96 total datapoints per enzyme). Depending on enzyme efficiency and assay sensitivity, even lower enzyme amounts or reaction volumes may be used, expanding the data that can be collected from a single-well enzyme purification. The enzymes selected for this study represent many of the highest-performing PET hydrolases reported to date, but activity assay conditions and reported data vary significantly across studies^67^. In this report, we present a side-by-side comparison in the same assay setup to identify the highest performers under equivalent conditions. However, these data do not necessary represent the highest activity achievable by each enzyme in its own optimized conditions^67^. For example, some PET hydrolases are suggested to have improved activity in the presence of Ca^2+^, which was a factor not considered in this study^47,55^. We intended these data to give information about the relative activities of these PET hydrolases across a broad range of conditions and showcase the wealth of data that can be achieved from these small-scale enzyme purifications.

We investigated a pH range from 4.5 to 9.5, to identify enzymes that maintain highest activity at low pH. Most large-scale enzymatic PET depolymerization reactions occur near neutral pH with pH control through base addition to prevent enzyme inactivation due to the pH drop resulting from acidic product release. Considering recent techno-economic analysis and life cycle assessment demonstrating the significant contribution of base addition to the economic and environmental impacts of this process, identifying enzymes that maintain activity at low pH becomes valuable in avoiding the need for pH control^68,69^. PET amorphization is another energetically intensive step in the enzymatic PET depolymerization process, leading to our investigation of both amorphous and crystalline PET substrates to identify enzymes with superior activity on crystalline substrates. LCC-A2, a variant of LCC-ICCG optimized for PET binding, emerged as the top-performing enzyme across most reaction conditions explored in this study.

The evolving landscape of protein engineering and the increasing demand for tailored enzymes necessitate a transition from traditional, labor-intensive protein purification methods to small-scale, high-throughput automated approaches. To promote accessibility and collaboration, we provided open-source code for this expression and purification protocol, allowing other research groups the ability to modify and improve the method. The Supplementary Information is designed to make this high-throughput platform accessible to early career researchers, offering detailed protocols that elucidate steps that are often otherwise assumed knowledge in biochemistry research. With increased access to automation, the field of enzyme engineering is poised for rapid expansion in the discovery of novel and improved enzymes, contributing to new biological insights and discoveries.

## Methods

### Transformation

Genes were codon optimized, synthesized, and cloned into pCDB179 (gifted to Addgene by Christopher Bahl, #91960) by Twist Biosciences. If codon optimized sequences for *E. coli* expression were available in the literature for individual variants, those were used preferentially. All sequences are available in Supplementary File 1, and all plasmids have been deposited in Addgene (https://www.addgene.org/Gregg_Beckham/). Constructs consisted of an N-terminal 10xHis tag on the SUMO protein (Smt3) fused to the N-terminus of the target protein. Upon receipt, plasmid plates (with supplied plasmid amounts ranging from ~ 800–1100 ng) were centrifuged to pellet the lyophilized powders, then resuspended in 80 µL nuclease free water to approximately 8–11 ng/µL. 50 µL of chemically competent C41(DE3) *E. coli* cells prepared with the Zymo Mix and Go Transformation Kit (Zymo T3001) were added to each well of a sterile, pre-chilled 96-deep-well plate (NEST 503501). 5 µL of the 10 ng/µL plasmid solution was added to each well, the mixture gently mixed by shaking, then incubated on ice for 10 min. Then 150 µL of media was added, followed by a 1 h outgrowth step at 37 °C, 350 rpm (19 mm orbit). Then 150 µL of media supplemented with 2X antibiotic (100 ug/mL kanamycin) was added and growth continued for two overnights (~ 40 h) at 30 °C, 350 rpm (19 mm orbit). To store transformants as glycerol stocks, equal volume (300 µL) sterile 60% glycerol in water was added to each well and the plate stored at − 80 °C.

### Expression

Two mL of Overnight Express™ Instant TB autoinduction media (Novagen 71491) sterilized by microwave irradiation and supplemented with 2X antibiotic and 1X trace metals (Teknova T1001) in each well of a 24-deep-well plate (Thomson Instrument Company 931568) was inoculated with 20 µL of starter culture. Starter culture consisted of either transformants grown for two overnights to achieve saturation as described above or subcultured glycerol stocks. This second method was achieved by fully thawing the glycerol stock plate and inoculating 200 µL LB with 1X antibiotic with 10 µL of thawed glycerol stock then growing overnight at 30 °C, 350 rpm (19 mm orbit) or 900 rpm (3 mm orbit). Inoculated autoinduction cultures were sealed with breathable seals (AeraSeal Adhesive Sealing Film) and grown at 37 °C for 2–3 h then 18 °C for two overnights at 300 rpm (19 mm orbit).

### Lysis

Cells were sedimented by centrifugation at 2500 rpm (1862 × *g*), 4 °C, for 10 min and the supernatant is discarded by inverting the plate with a rapid and smooth motion to avoid spillover between wells. 1.5 mL of Lysis Buffer (20 mM TRIS pH 8.0, 300 mM NaCl, 5 mM imidazole, 1% *n*-octyl β-D-glucopyranoside supplemented with 0.1 mg/mL DNaseI and 1 mg/mL lysozyme) was added to each well. Cells were resuspended by shaking at 18 °C, 300 rpm (19 mm orbit) for 1 h.

### Purification

8 mL of Ni-charged magnetic beads (Genscript L00295) were washed 3 × with 50 mL of Wash Buffer (20 mM TRIS pH 8.0, 300 mM NaCl, 5 mM imidazole) to remove the ethanol storage solution then resuspended in 8 mL Wash Buffer. 70 µL of this magnetic bead suspension was added to each well and plates shaken at 18 °C, 250 rpm for 2 h. The magnetic beads were then pulled down and the supernatant discarded. The magnetic beads were then resuspended in 300 µL of Wash Buffer and transferred to a 96 well plate. The magnetic beads were washed 3 × with 300 µL Wash Buffer, and 2 × with 300 µL Cleavage Buffer (20 mM TRIS pH 8.0, 300 mM NaCl) and were resuspended in 300 µL Cleavage Buffer to which 30 µL of 0.75 mg/mL His-tagged *Cth* SUMO protease (produced in-house, protocol detailed in the Supplementary Material and Methods) was added^30^. The cleavage progressed for 3–4 h at 1200 rpm (3 mm orbit) on the OT-2 Heater-Shaker Module at room temperature then stored static overnight at 4 °C. The supernatant was then transferred off the magnetic beads to a fresh plate.

### Quantification

Concentration was measured using the Pierce Rapid Gold BCA Protein Assay Kit using 10 µL of sample and 200 µL of BCA reagent (50:1 reagent A to reagent B). The assay was incubated at room temperature for 25 min then the absorbance at 480 nm was recorded. Each plate contained the calibration BSA solutions prepared in the same buffer as the samples in concentrations ranging from 0 – 2 mg/mL.

### Normalization

The concentrations of the purified proteins were normalized by dilution. If the concentration was under 0.1 mg/mL, approximately the limit of detection for the concentration quantification assay, no dilution was performed. If above 0.1 mg/mL, the concentration was diluted to 0.1 mg/mL, unless this step would exceed the volume of the 2 mL well, in which case the concentration was diluted to 0.3 mg/mL. The dilution buffer used was 20 mM TRIS pH 8.0, 300 mM NaCl.

### Differential scanning fluorimetry

45 µL from each normalized well was transferred to a PCR plate (Bio-Rad HSP9601) containing 5 µL in each well of a 5X solution of Sypro Orange Dye (ThermoFisher Scientific S6650) in matching buffer to the sample. The plate was sealed (ThermoFisher Scientific 4311971) and fluorescence was measured on a Bio Rad CFX96 Touch Real-Time PCR instrument with the following program: 1. 25 °C: 0:15; 2. 25 °C: 0:31; 3. 25 °C: 0:15 (+ 0.3 °C/cycle, ramp 0.3 °C/sec); 4. Plateread, 5. Go to 3, 250X.

### Activity assay

2 mL deep-well 96-well plates were loaded with 3.5 mg of amorphous PET film (Goodfellow ES30-FM-000145) or crystalline PET powder (Goodfellow ES30-PD-006031). The amorphous film was cut using a Cricut Maker, into 0.34 × 0.34 cm squares (https://design.cricut.com/landing/project-detail/64d402ee263e5d6d3d2614b1) and loaded manually into each well aided by tweezers and antistatic gloves (Uline S-15357). The crystalline powder was added using a Powdernium Automated Powder Dosing System (Symyx). 475 µL of buffer containing 50 mM of NaCitrate (pH 4.5, 5.5), NaPhosphate (pH 6.5, 7.5), or glycine (pH 8.5, 9.5) was added. Buffers were prepared to the desired pH at room temperature. The plates were sealed Nunc Aluminum Seal Tape (Thermo Scientific 232698) and incubated at the desired assay temperature (either 60 or 70 °C) for 2 h. The enzymes to be assayed were pooled from multiple wells if necessary, normalized to 0.1 mg/mL, and reordered in a fresh 96-well plate into two columns for addition to the assays via multichannel pipette. 25 µL of enzyme (2.5 µg) was added to each well with the blank containing the same buffer as the enzyme solutions. The plates were sealed with aluminum heat sealing foil (Azenta 4ti-0535) using a Vitl VTS Variable Temperature Microplate Sealer for 6 s at 165 °C. The edges were taped with autoclave tape to further reduce evaporation. Plates were incubated at the desired temperature and mixed via shaking at 250 rpm in a 19 mm orbit incubator for either 2 or 24 h, at which point they were removed, allowed to cool for 30 min, then stored at − 20 °C.

### UV–Vis analysis

After thawing, the assays containing crystalline power were filtered through MultiScreen_HTS_ GV Filter Plates (Millipore MSGVN2210) using centrifugation (950 × *g,* 6 min) assisted by receiver plate collars (Pall Life Sciences 5225). The assays containing amorphous film were moved directly to UV–Vis analysis. 100 µL of sample was transferred from the assays to a UV-transparent flat bottom plate (Greiner 655801) and read at 260 and 280 nm on a BioTek Synergy H1. Blank subtraction was performed against the no-enzyme control for each pH. The following extinction coefficients were calculated from calibration curves for TPA (ε_260_ = 4.588 mM^-1^cm^-1^, ε_280_ = 1.300 mM^-1^cm^-1^) and MHET (ε_260_ = 6.519 mM^-1^cm^-1^, ε_280_ = 1.767 mM^-1^cm^-1^) (Supplementary Fig. 23). An average of TPA and MHET extinction coefficients for each wavelength was used in the calculations of product concentration, as previously described^64^. For samples with high product concentrations that resulted in detector saturation, a dilution was performed on the entire well, the solution mixed, and then another read was performed on a 100 µL aliquot.

### HPLC analysis

TPA, MHET, and BHET were analyzed by ultra-high performance liquid chromatography (UHPLC) as previously detailed^12^. Briefly, samples and standards were analyzed by UHPLC coupled with diode array detection (DAD). Chromatographic separation was accomplished using a mobile gradient consisting of 20 mM phosphoric acid and methanol and a Zorbax Eclipse Plus C18 Rapid Resolution HD analytical column. A quantification wavelength of 240 nm was used to construct calibration curves for quantitation of each analyte of interest.

### Supplementary Information

Supplementary Information.

Source Data.

Supplementary File 1.

## Data Availability

Data are provided within the manuscript or supplementary information files.
