# Supplementary material for: Enabling high-throughput enzyme discovery and engineering with a low-cost, robot-assisted pipeline
Source: Sci Rep. 2024 Jun 24;14:14449. doi: 10.1038/s41598-024-64938-0 (PMC11196671; doi:10.1038/s41598-024-64938-0)
Supplement: Supplementary file 1 — Supplementary Information. [file 41598_2024_64938_MOESM1_ESM.pdf]

## Supplementary Information for:

# Enabling high-throughput enzyme discovery and engineering with a low-cost, robot-assisted pipeline

Brenna Norton-Baker,[1,2,3] Mackenzie C.R. Denton,[1,2] Natasha P. Murphy,[1,2] Benjamin Fram,[4] Samuel Lim,[4] Erika Erickson,[1,2] Nicholas P. Gauthier,[4,5*] Gregg T. Beckham[1,2,3*]

[1]Renewable Resources and Enabling Sciences Center, National Renewable Energy Laboratory, Golden, CO, USA
[2]BOTTLE Consortium, Golden, CO, USA
[3]Agile BioFoundry, Emeryville, CA, USA
[4]Department of Systems Biology, Harvard Medical School, Boston, MA, USA
[5]Department of Data Sciences, Dana-Farber Cancer Institute, Boston, MA, USA

* Correspondence: gregg.beckham@nrel.gov; nicholas.gauthier.research@gmail.com

**Table of Contents**


**Supplementary Materials and Methods ....3**
**Supplementary Tables and Figures ....4**
- **Supplementary Table 1. Recommended equipment and suggested substitutions .... 4**
- **Supplementary Table 2. Labware definitions and custom labware files. .... 4**
- **Supplementary Table 3. Buffers and reagents compositions. .... 5**
- **Supplementary Table 4. Transformation protocol .... 6**
- **Supplementary Table 5. Inoculation protocol. .... 7**
- **Supplementary Table 6. Lysis protocol .... 8**
- **Supplementary Table 7. Transfer protocol from four 24-well plates to one 96-well plate .... 9**
- **Supplementary Table 8. Purification protocol. .... 10**
- **Supplementary Table 9. Bicinchoninic acid (BCA) setup protocol .... 11**
- **Supplementary Table 10. Concentration normalization protocol .... 11**
- **Supplementary Table 11. Differential scanning fluorimetry (DSF) setup protocol .... 11**
- **Supplementary Figure 1. Red fluorescent protein (RFP) .... 12**
- **Supplementary Figure 2. Construction of the home-built magnetic module .... 13**
- **Supplementary Figure 3. Expression yields .... 14**
- **Supplementary Table 13. Summary of intact mass spectrometry data for selected enzymes. .... 15**
- **Supplementary Figure 4. Intact mass spectrometry for LCC-ICCG .... 16**
- **Supplementary Figure 5. Intact mass spectrometry for LCC-ICCG RIP .... 17**
- **Supplementary Figure 6. Intact mass spectrometry for LCC-ICCG DAQI .... 18**
- **Supplementary Figure 7. Intact mass spectrometry for LCC-ICCG I6M .... 19**
- **Supplementary Figure 8. Intact mass spectrometry for LCC-A2 .... 20**
- **Supplementary Figure 9. Intact mass spectrometry for SfCut .... 21**
- **Supplementary Figure 10. Intact mass spectrometry for Cut190*SS .... 22**
- **Supplementary Figure 11. Intact mass spectrometry for CaPETaseM9 .... 23**
- **Supplementary Figure 12. Intact mass spectrometry for TfCut2 .... 24**
- **Supplementary Figure 13. Intact mass spectrometry for $TfCut2_{S121P/D174S/D204P}$ .... 25**
- **Supplementary Figure 14. Intact mass spectrometry for $TfCut2_{L32E/S113E/T237Q}$ .... 26**
- **Supplementary Figure 15. Intact mass spectrometry for HotPETase .... 27**
- **Supplementary Figure 16. Intact mass spectrometry for Z1-PETase .... 28**
- **Supplementary Figure 17. SDS-PAGE post-purification of all enzymes studied .... 29**
- **Supplementary Figure 18. SDS-PAGE analysis of multiple stages of purification, .... 30**
- **Supplementary Figure 19. Differential Scanning Fluorimetry (DSF) data .... 31**
- **Supplementary Figure 20. Enzyme activity data measured at 24 h .... 32**
- **Supplementary Figure 21. UV-Vis analysis compared to HPLC analysis .... 33**
- **Supplementary Figure 22. Enzyme activity data measured at 2 h .... 34**
- **Supplementary Figure 23. UV-Vis spectroscopy calibration curves .... 35**

## Supplementary Materials and Methods

*Intact protein mass spectrometry*

Intact protein mass spectrometry was performed at University of Colorado Boulder Mass Spectrometry Facility. Protein samples were directly injected onto a 2.1 x 5 mm Acquity UPLC BEH300 C4, 1.7 μm VanGuard Pre-Column (Waters), using a Waters Acquity classic UPLC. After sample loading, the column was washed at 0.2 mL/minute for 3 minutes with 3% (v/v) acetonitrile (ACN), then eluted with a gradient from 3% to 85% (v/v) ACN in 3 minutes then to 95% (v/v) ACN in 0.5 minutes. Detection occurred using a Synapt G2 Q-Tof mass spectrometer (Waters). Positive ES resolution mode was used for precursor mass spectra (MS1) from 200 to 2,500 m/z with: 3.0 kV capillary voltage, 80 °C source temperature, 150 °C desolvation temperature, and 550 L/h nitrogen desolvation gas flow. Deconvolution was performed using Mass Lynx v4.2 Maximum Entropy 1.

*Sodium dodecyl-sulfate polyacrylamide gel electrophoresis*

A 15 μL sample of protein was mixed with 15 μL of 2X LDS Sample Buffer (Genscript M00676-10) supplemented with 1 mM dithiothreitol (DTT). Samples were heated at 90 °C for 2-3 min. Samples were run on a 15% polyacrylamide gel at 200 V for 35 min and stained using Coomassie blue dye with the Pierce Power Stainer system.

*Cth SUMO protease expression and purification*

Chemically competent C41(DE3) *E. coli* cells prepared with the Zymo Mix and Go Transformation Kit were transformed with pCDB302 (gifted to Addgene by Christopher Bahl, #113673). Expression cultures containing 200 mL autoinduction media (Overnight Express Instant TB Medium, Novagen 71491) supplemented with 1X trace metals (Teknova T1001) and 100 μg/mL kanamycin in 1 L baffled flasks were inoculated with 750 μL of overnight saturated starter culture and grown at 37 °C, 250 rpm for 2-3 h followed by 25 °C, 250 rpm for 24 h. Cells were harvested by centrifugation, the supernatant discarded, and the cell pellets flash frozen in liquid nitrogen. Cells were resuspended in Wash Buffer (20 mM Tris pH 8, 300 mM NaCl, 5 mM imidazole) supplemented with 0.1 mg/mL DNaseI and 1 mg/mL lysozyme then sonicated for 2 min process time with 1 s on and 1 s off at 50% amplitude (QSonica Q700). The lysate was clarified by centrifugation and the supernatant filtered through 0.45 μm syringe filters before purification via Ni-affinity chromatography. The sample was loaded on a 5 mL HisTrap column (Cytiva 17524801), washed with Wash Buffer, then eluted with Elution Buffer (20 mM Tris pH 8, 300 mM NaCl, 400 mM imidazole. The sample was dialyzed into Storage Buffer (50 mM Tris pH 8, 200 mM NaCl). After dialysis, precipitation was pelleted and the supernatant isolated. The sample was then supplemented to reach 1 mM DTT, 1 mM EDTA, and 5% glycerol and brought to 3.5 mg/mL for storage. The samples were aliquoted, flash frozen, and stored at -80 °C. Yields averaged approximately 30 mg/L autoinduction culture.

## Supplementary Tables and Figures

**Supplementary Table 1.** Recommended equipment and suggested modifications/substitutions.

| **Expression and purification** | | |
|---|---|---|
| Recommended equipment | • Shaker-incubator (19 mm orbit or similar)<br>• Multichannel pipette(s) (electronic repeater pipettes recommended)<br>• Centrifuge with plate rotor<br>• Plate reader<br>• OT-2<br>• OT-2 pipettes:<br>  ○ 1 mL single channel pipette<br>  ○ 20 $\mu$L multichannel pipette<br>  ○ 300 $\mu$L single channel pipette<br>  ○ 300 $\mu$L multichannel pipette<br>• OT-2 Magnetic Module GEN2<br>• OT-2 Heater Shaker Module<br>• OT-2 Temperature Module GEN2 | |
| **Thermostability characterization and activity assay setup** | | |
| Additional recommended equipment | • Real-time PCR or nanoDSF instrument<br>• Plate heat sealer | |
| **Modifications/substitutions** | | |
| Equipment | Modification | Comments |
| Autoclave for media preparation | Substitute with microwave | For the Overnight Express Instant TB Medium, the microwave method as detailed in the manufacturer's protocol was preferred for rapid media sterilization. |
| OT-2 Temperature Module GEN2 | Manual transformation | Chill transformation plate on ice and use a multichannel pipette for the transformation step. |
| OT-2 Heater Shaker Module | Use a separate plate shaker, or avoid the need to shake magnetic beads in 96-well plate | For purification: use *Pipetting only purification* |

**Supplementary Table 2.** Labware definitions and custom labware files.

| **Opentrons Labware Library** | | |
|---|---|---|
| Description | Source | API name |
| 2.0ml 96 Well Deep Well Plates Square Well, V-bottom | NEST 503501 | nest_96_wellplate_2ml_deep |
| NEST 12 Well Reservoir 15 mL, V-bottom | NEST 360102 | nest_12_reservoir_15ml |
| NEST 1 Well Reservoir 195 mL, V-bottom | NEST 360103 | nest_1_reservoir_195ml |
| Bio-Rad 96 Well Plate 200 $\mu$L PCR, V-bottom | Bio-Rad HSP9601 | biorad_96_wellplate_200ul_pcr |
| Opentrons 96 Tip Rack 1000 $\mu$L | Opentrons 999-00010 | opentrons_96_tiprack_1000ul |
| Opentrons 96 Filter Tip Rack 200 $\mu$L | Opentrons 999-00081 | opentrons_96_filtertiprack_200ul |
| Opentrons 96 Tip Rack 300 $\mu$L | Opentrons 999-00009 | opentrons_96_tiprack_300ul |
| Opentrons 96 Tip Rack 20 $\mu$L | Opentrons 999-00007 | opentrons_96_tiprack_20ul |
| **Custom Labware** | | |
| Description | Source | API name (with link to download) |
| Twist V-bottom 200 $\mu$L 96-well plates | Twist Biosciences | twist_96_wellplate_400ul |
| Thomson Instrument Company 24-Well Plate, 10.4mL, Square Well, Round Bottom | Thomson Instrument Company 931568 | thomsoninstrument_24_wellplate_10400ul |
| Thomson Instrument Company 24-Well Plate, 10.4mL, Square Well, Round Bottom on top of a NEST 2.0ml 96 Well Deep Well Plates Square Well, V-bottom loaded with bar magnets (Home-built magnetic module) | Assembled in-house | thomsoninstrument_24_wellplate_10400ul_on_homebuilt_magmod |

**Supplementary Table 3.** Buffers and reagents compositions.

| Name | Composition |
|---|---|
| Lysis Buffer | 20 mM TRIS pH 8.0,<br>300 mM NaCl,<br>5 mM imidazole,<br>1% *n*-octyl β-D-glucopyranoside,<br>0.1 mg/mL DNaseI,<br>1 mg/mL lysozyme |
| Wash Buffer | 20 mM TRIS pH 8.0,<br>300 mM NaCl,<br>5 mM imidazole |
| Cleavage Buffer | 20 mM TRIS pH 8.0,<br>300 mM NaCl |
| Kanamycin 2000X | 100 mg/mL kanamycin in water |
| Trace Metals Mixture 1000X (Teknova T1001) | 13.52 mg/mL Ferric Chloride,<br>2.94 mg/mL Calcium Chloride,<br>1.98 mg/mL Manganese Chloride,<br>2.88 mg/mL Zinc Sulfate,<br>476 $\mu$g/mL Cobalt Chloride,<br>341 $\mu$g/mL Cupric Chloride,<br>455 $\mu$g/mL Nickel Chloride,<br>489 $\mu$g/mL Sodium Molybdate,<br>346 $\mu$g/mL Sodium Selenite,<br>124 $\mu$g/mL Boric Acid |

**Supplementary Table 4.** Detailed transformation protocol with linked OT-2 scripts.

<table>
<tr><th colspan="2">1 - Transformation</th></tr>
<tr><td>1.</td><td>Pre-chill a 96-deep-well plate at -20 °C.</td></tr>
<tr><td>2.</td><td>Prepare plasmids:<br>a. Centrifuge to pellet lyophilized powder or liquid.<br>b. Resuspend or dilute to achieve plasmid concentration ~10 ng/μL (this can be very flexible).</td></tr>
<tr><td>3.</td><td>Thaw competent cells on ice. (Prepared with Zymo Mix and Go Transformation Kit)</td></tr>
<tr><td>4.</td><td>Transform</td></tr>
<tr><td></td><td>Option 1: Manual Transformation<br>1. Transfer 50 μL of competent cells to each well.<br>2. Add 5 μL of desired plasmid to each well.<br>3. Incubate on ice 5-10 min.</td></tr>
<tr><td></td><td><u>OT-2</u> - Option 2: Plate-to-plate transformation<br><br>This OT-2 protocol uses a multichannel pipette to transfer the plasmids from the plasmid library plate to the transformation plate in a direct plate stamp. This protocol requires the OT-2 Temperature Module GEN2, p20 multichannel pipette.<br><br>Link: Plate-to-plate transformation<br><br>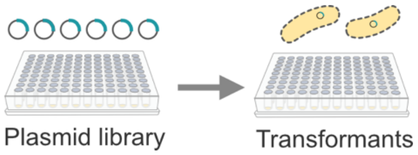
</td></tr>
<tr><td></td><td><u>OT-2</u> - Option 3: Cherry-picking transformation<br><br>This OT-2 protocol uses a single channel pipette to transfer plasmids from up to 3 different plasmid plates to the transformation plate. An Excel template is provided to specify the source and destination wells. This protocol requires the OT-2 Temperature Module GEN2 and the p20 single channel pipette.<br><br>Link: Cherry-picking transformation<br><br>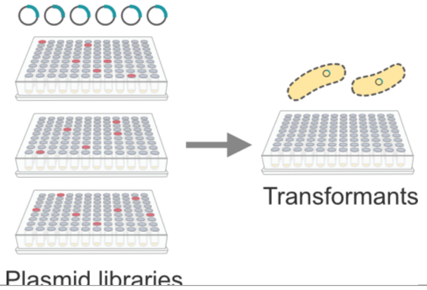
</td></tr>
<tr><td>5.</td><td>Add 150 μL LB without antibiotics.</td></tr>
<tr><td>6.</td><td>Seal with breathable seal and shake at 30 °C for 1 h, 350 rpm.*</td></tr>
<tr><td>7.</td><td>Add 150 μL LB with 2X antibiotics.</td></tr>
<tr><td>8.</td><td>Seal with breathable seal and grow overnight for two nights at 30 °C, 350 rpm.*</td></tr>
<tr><td colspan="2">Notes</td></tr>
<tr><td colspan="2">*This step is optimized for a standard shaker (19 mm orbit). If using a plate shaker (3 mm orbit) timing may need to be adjusted.</td></tr>
<tr><td colspan="2">To save transformants: Save the transformation overnight plate as a glycerol stock by adding equal volume of a 60% sterile glycerol/$H_2O$ solution. Store directly at -80 °C.<br><br>To use the glycerol stock, fully thaw plate at 37 °C then mix well. Scraping frozen stocks using a multichannel pipette did not yield consistent results in successfully inoculating all wells of the overnight cultures. Use the thawed plate to inoculate an overnight starter culture plate by adding 10 μL to 200 μL LB supplemented with antibiotic. We have used a glycerol stock > 3 times; however, repeated freeze-thaw cycles will decrease cell viability.</td></tr>
</table>

**Supplementary Table 5.** Detailed inoculation and expression protocol with linked OT-2 scripts.

| **2 – Inoculation and Expression** | |
|---|---|
| 1. | Prepare autoinduction media following manufacturer's instructions: |
| | a. 15 g Overnight Express™ Instant TB Medium, 3.75 mL 60% glycerol, bring to 250 mL with water in a 500 mL bottle<br>b. Microwave until mixture is boiling for 30 s total.<br>c. Cool to room temperature.<br>d. Supplement to reach necessary antibiotic levels (for kanamycin in autoinduction expressions, 2X is recommended) and 1X trace metals. |
| 2. | Add 2 mL of autoinduction media to each well of four 24-deep-well plates. |
| 3. | Inoculate |
| | **OT-2 - Option 1: Plate-to-plate inoculation**<br><br>This OT-2 protocol uses a single pipette to transfer the inoculum from the overnight starter plate to the expression plates. This protocol requires the OT-2 p300 single channel pipette.<br><br>Link: Plate-to-plate inoculation<br><br>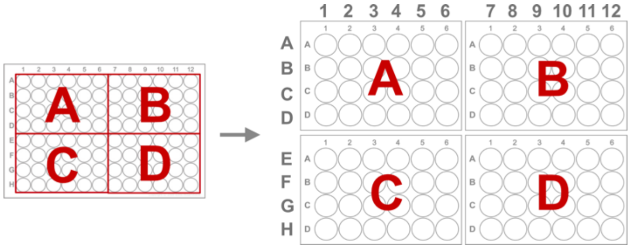 |
| | **OT-2 - Option 2: Row-swap inoculation** (must be reversed later in transfer step)<br><br>This OT-2 protocol uses a half-loaded multichannel pipette to transfer the inoculum from the overnight starter plate to the expression plates. This protocol requires the OT-2 p300 multichannel pipette.<br><br>Link: Row-swap inoculation<br><br>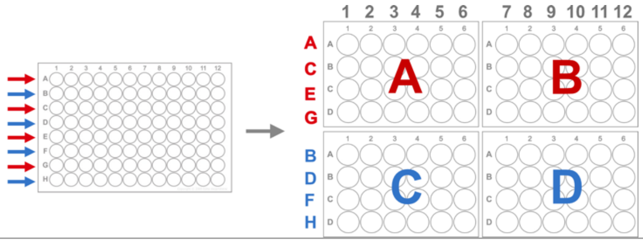 |
| | **OT-2 - Option 3: Cherry-picking inoculation**<br><br>This OT-2 protocol uses a single channel pipette to transfer the inoculum from up to 3 different overnight starter plates to the expression plates. An Excel template is provided to specify the source and destination wells. This protocol requires the OT-2 p300 single channel pipette.<br><br>Link: Cherry-picking inoculation<br><br>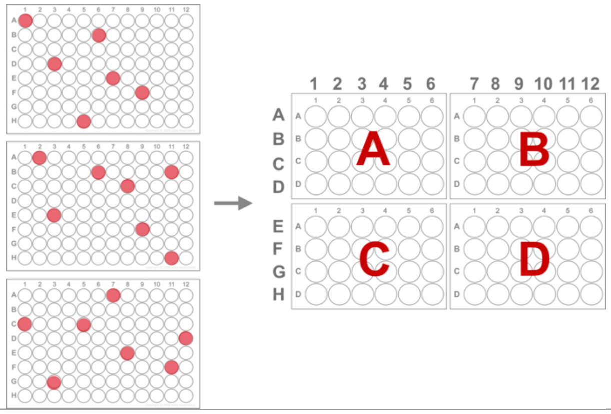 |
| 4. | Seal plates with breathable seals and shake 37 °C, 300 rpm for minimum 2 h. |
| 5. | Shake at 18 °C, 300 rpm overnight for two nights. |

**Supplementary Table 6.** Detailed lysis protocol with linked OT-2 scripts.

| **3 – Lysis** | |
|---|---|
| 1. | Centrifuge plates at 2500 rpm (1862 × *g*), 4 °C, 10 min to pellet cells. |
| 2. | Discard supernatant by plate inversion – used a rapid, smooth motion to avoid spillover. |
| 3. | Add 1.5 mL of Lysis Buffer to each well (manually or OT-2 Lysis buffer addition) |
| 4. | Shake plates at 18 °C, 300 rpm (19 mm orbit) for 1 h to resuspend cells. |
| 5. | Wash 8 mL of Ni-charged magnetic beads 3x with 50 mL Wash Buffer and resuspend in final volume of 8 mL. |
| 6. | Add 70 μL of the washed magnetic beads to each well. The magnetic beads settle quickly, resuspend beads before each transfer to ensure even addition. |
| 7. | Shake plates at 18 °C, 250 rpm (19 mm orbit) for minimum 2 h for binding. |
| 8. | Remove supernatant |
| | **Option 1: Manual aspiration**<br>1. Pull down magnetic beads using the OT-2 magnetic module or the home-built magnetic module.<br>2. Using a large volume multichannel pipette (1.2 mL), aspirate the supernatant and discard.<br>3. Add 300 uL Wash Buffer to each well. |
| | **OT-2 - Option 2: 1-plate aspiration**<br><br>This OT-2 protocol uses a multichannel pipette to aspirate the supernatant and add Wash Buffer for one 24-well plate at a time. This protocol requires the OT-2 Magnetic Module and a p300 multichannel pipette.<br><br>Link: 1-plate aspiration |
| | **OT-2 - Option 3: 4-plate aspiration**<br><br>This OT-2 protocol uses a multichannel pipette to aspirate the supernatant and add Wash Buffer for four 24-well plates at a time. This protocol requires four home-built magnetic modules (Supplementary Figure 1) and a p300 multichannel pipette.<br><br>Link: 4-plate aspiration |
| 9. | Briefly shake plates to resuspend magnetic beads. |

**Supplementary Table 7.** Detailed transfer protocol from four 24-well plates to one 96-well plate with linked OT-2 scripts.

**4 – Transfer**

1. Transfer to 96-well plate

**Option 1: Manually transfer**

1. Manually transfer each well from four 24-well plates back to the corresponding well in one 96-well plate.

**<u>OT-2</u> - Option 2: Plate-to-plate transfer**

This OT-2 protocol uses a single channel pipette to transfer the magnetic beads from four 24-well plates to one 96-well plate. This protocol requires a p1000 single channel pipette.

Link: [Plate-to-plate transfer]

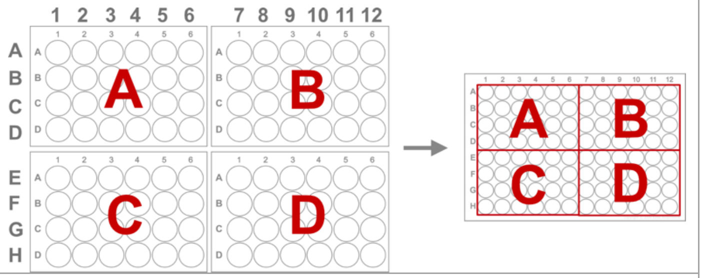


**<u>OT-2</u> - Option 3: Row-swap transfer**

This OT-2 protocol uses a half-loaded multichannel pipette to transfer the magnetic beads from four 24-well plates to one 96-well plate. This protocol requires a p300 multichannel pipette.

Link: [Row-swap transfer]

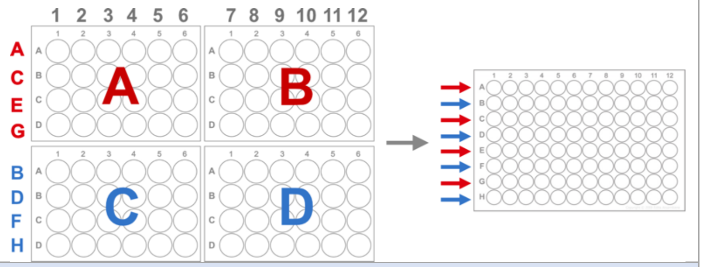


**Notes**

These OT-2 protocols rely on very accurate labware calibration to ensure the entire contents of the well are aspirated from the well bottom.

**Supplementary Table 8.** Detailed purification protocol with linked OT-2 scripts.

| **5 – Purification** | |
|---|---|
| 1. | Purification |
| | **OT-2 - Option 1: Purification with shaker**<br><br>This OT-2 protocol uses a multichannel pipette to wash the magnetic beads and add the protease. This protocol requires an OT-2 Magnetic Module GEN2, an OT-2 Heater-Shaker Module, and a p300 multichannel pipette.<br><br>Link: Purification with shaker |
| | **OT-2 - Option 2: Pipetting-only purification**<br><br>This OT-2 protocol uses a multichannel pipette to wash the magnetic beads and add the protease. This protocol requires an OT-2 Magnetic Module GEN2 and a p300 multichannel pipette.<br><br>Link: Pipetting-only purification |
| 2. | Allow protease cleavage to proceed for 3-4 h at room temperature. *Optional:* Store at 4 °C overnight. |
| 3. | Transfer supernatant off magnetic beads |
| | **OT-2 - Transfer supernatant off magnetic beads with OT-2 Magnetic Module**<br><br>This OT-2 protocol uses a multichannel pipette to transfer the supernatant off the magnetic beads to a fresh plate. This protocol requires an OT-2 Magnetic Module GEN2 and a p300 multichannel pipette.<br><br>Link: Transfer off magnetic beads with OT-2 Magnetic Module |

**Supplementary Table 9.** Detailed bicinchoninic acid (BCA) setup protocol with linked OT-2 scripts.

| **6 – BCA Assay Setup** | |
|---|---|
| 1. | BCA Assay Setup |
| | **OT-2 – BCA Assay Setup**<br><br>This OT-2 protocol uses multichannel pipettes to combine the proteins samples and control samples with the BCA reagent. This protocol requires a p20 multichannel pipette and a p300 multichannel pipette.<br><br>Link: BCA assay setup |

**Supplementary Table 10.** Detailed concentration normalization protocol with linked OT-2 scripts.

| **7 – Concentration Normalization** | |
|---|---|
| 1. | Concentration Normalization |
| | **OT-2 – Concentration Normalization**<br><br>This OT-2 protocol uses a single channel pipette to dilute each well of the purified protein plate to the desired concentration(s). An Excel template is provided to import the starting concentrations. This protocol requires a p1000 single channel pipette and a p300 single channel pipette.<br><br>Link: Concentration normalization |

**Supplementary Table 11.** Detailed differential scanning fluorimetry (DSF) with Sypro Orange dye setup protocol with linked OT-2 scripts.

| **8 – DSF Setup** | |
|---|---|
| 1. | DSF Setup |
| | **OT-2 – DSF Setup**<br><br>This OT-2 protocol uses multichannel pipettes to combine the proteins samples with the Sypro Orange dye. This protocol requires a p20 multichannel pipette and a p300 multichannel pipette.<br><br>Link: DSF setup |

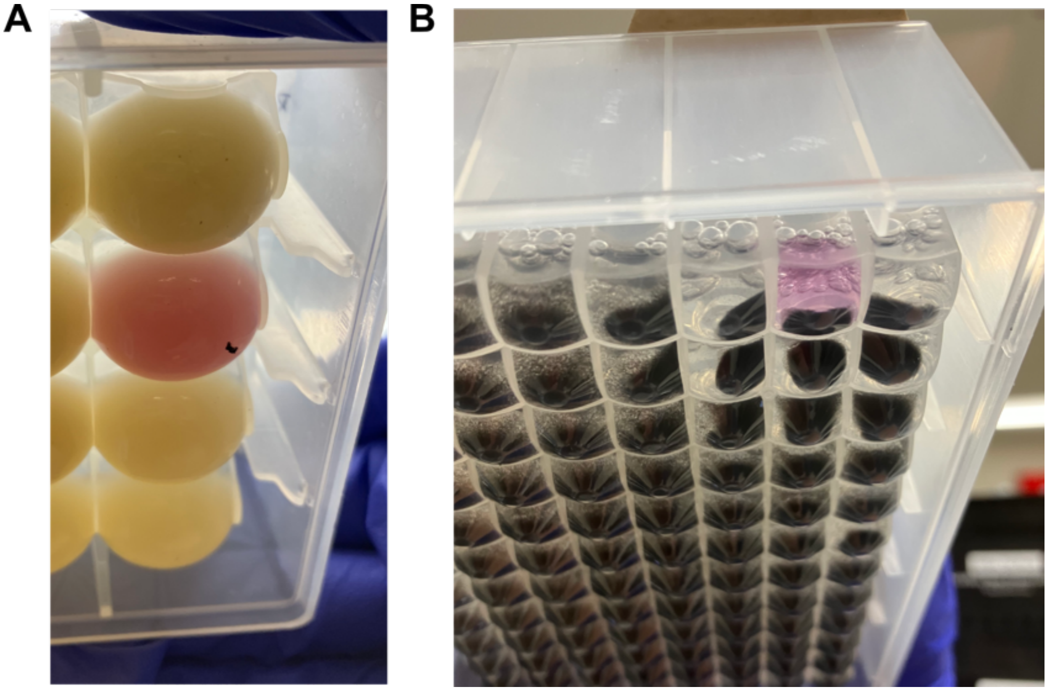

**Supplementary Figure 1.** Red fluorescent protein (RFP) as a visible reporter at different stages of the protocol. **(A)** RFP showed a bright pink color at the end of expression. **(B)** RFP released into the supernatant after protease cleavage off of the magnetic beads.

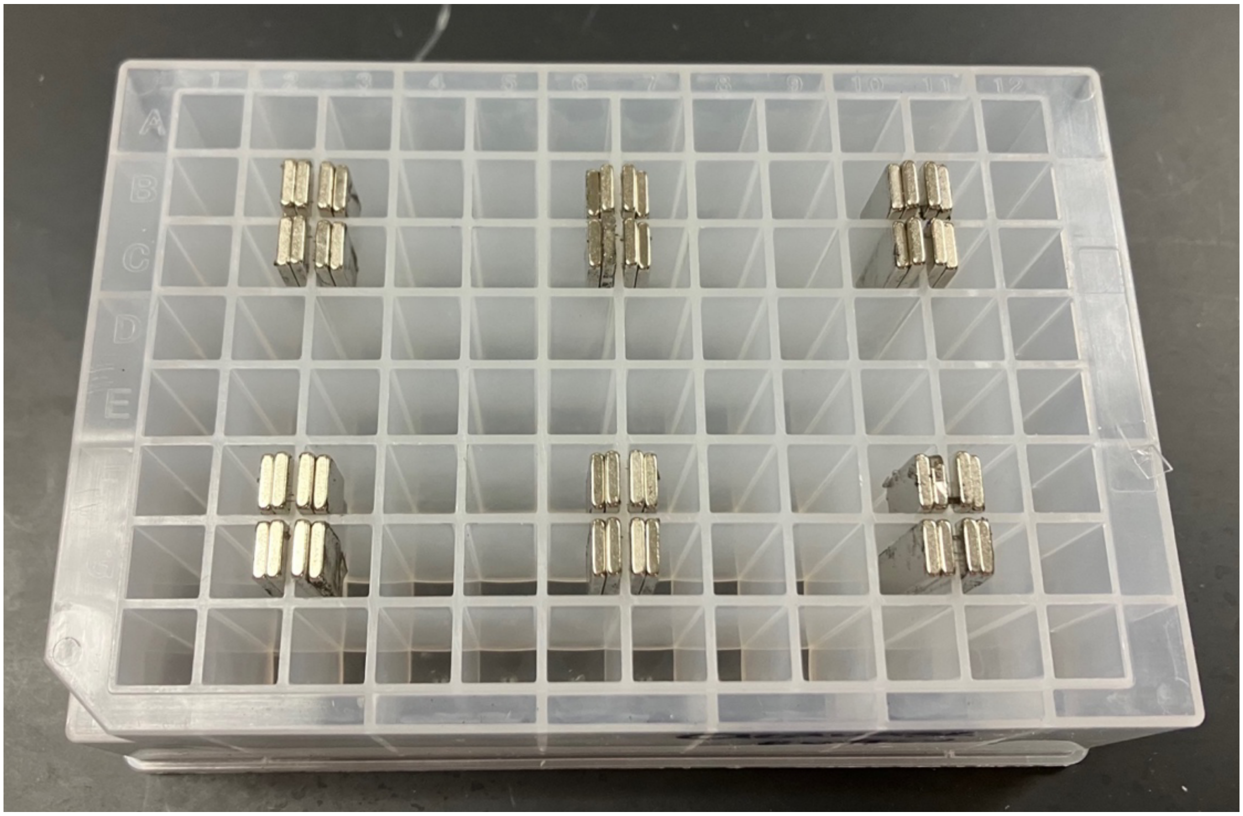


| Material | Source |
| --- | --- |
| 2.0ml 96 Well Deep Well Plates Square Well, V-bottom | NEST 503501 |
| Neodymium Block Magnet, 1" x 1/4" x 1/16" | K&J Magnetics Inc. BX041 |

**Supplementary Figure 2.** Construction of the home-built magnetic module for a 24-well plate is achieved by placing bar magnets in the positions shown in the image. The materials used are listed in the table.

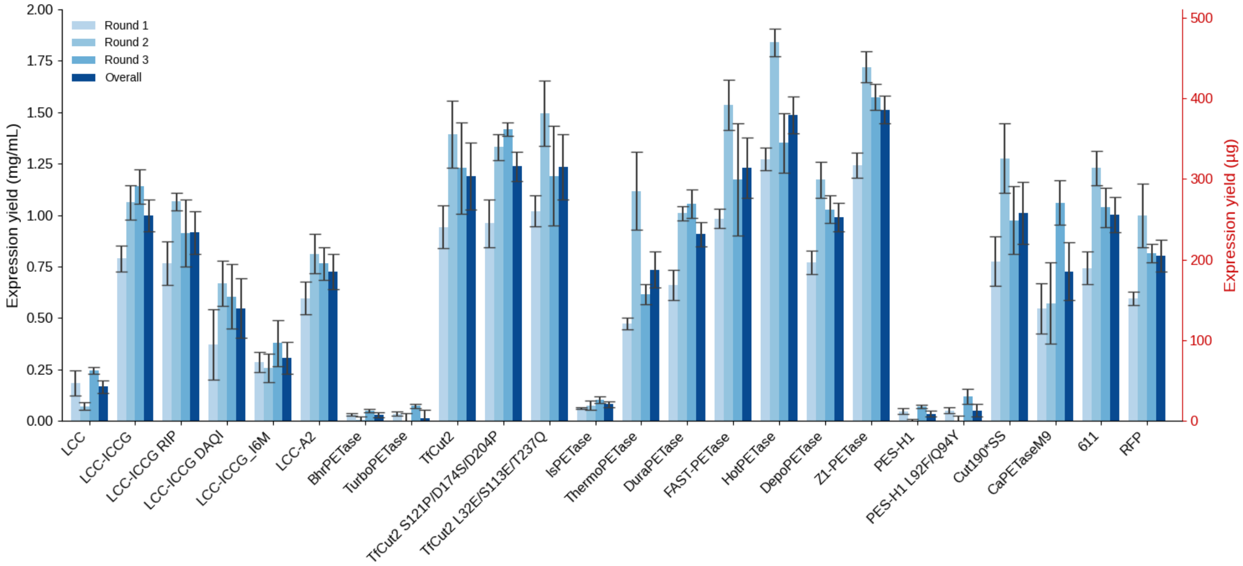


**Supplementary Figure 3.** Expression yields from three separate rounds expressed in mg/mL of final protein concentration and in total $\mu$g. Data are presented as mean values ± standard deviation ($n$=4 for each trial and $n$=12 for overall yield from all three trials).

**Supplementary Table 13.** Summary of intact mass spectrometry data for selected enzymes.

| Name | Expected Mass (Da) | Measured Mass (Da) |
|---|---|---|
| LCC-ICCG | 27638 | 27634 |
| LCC-ICCG RIP | 27720 | 27716 |
| LCC-ICCG DAQI | 27707 | 27703 |
| LCC-ICCG I6M | 27697 | 27693 |
| LCC-A2 | 27665 | 27661 |
| *Sf*Cut | 27893 | 27892 |
| Cut190*SS | 28608 | 28601 |
| *Ca*PETaseM9 | 27714 | 27714 |
| *Tf*Cut2 | 28229 | 28221 |
| *Tf*Cut2$_{S121P/D174S/D204P}$ | 28193 | 28198 |
| *Tf*Cut2$_{L32E/S113E/T237Q}$ | 28314 | 28313 |
| HotPETase | 27577 | 27570 |
| Z1-PETase | 27667 | 27659 |

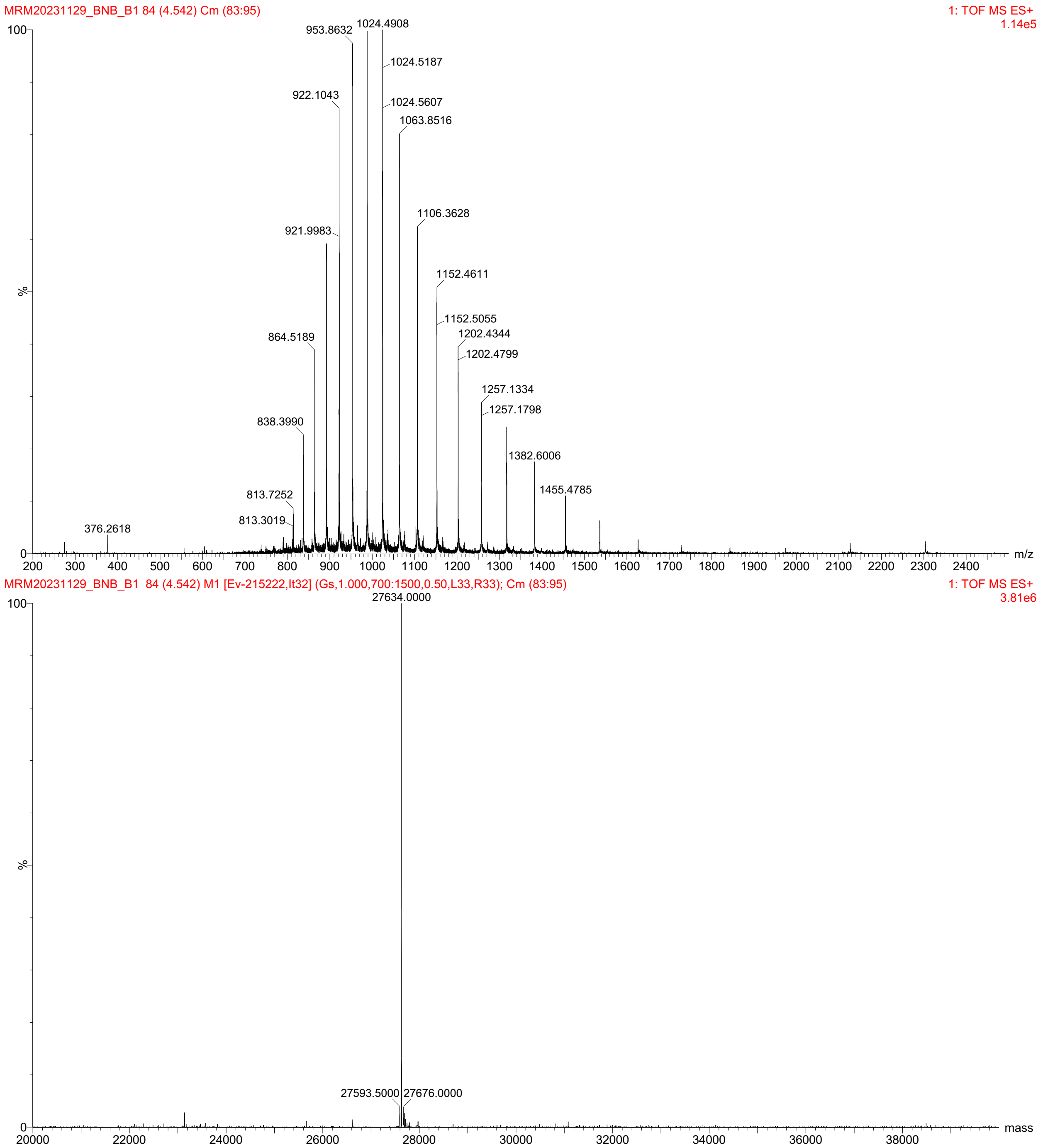


**Supplementary Figure 4.** Intact mass spectrometry for LCC-ICCG with the top showing the full charge ladder and the bottom showing the deconvoluted spectrum.

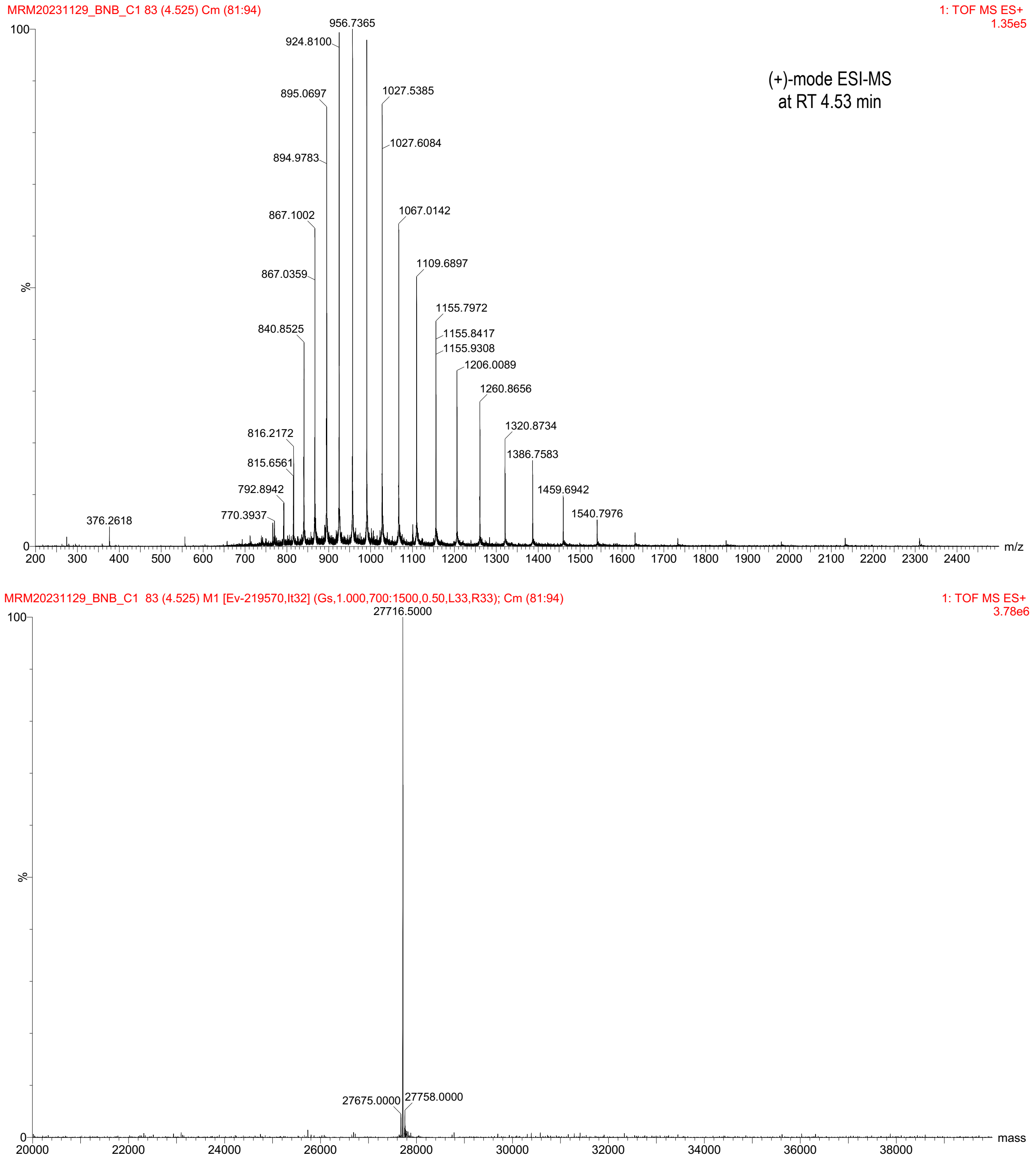


**Supplementary Figure 5.** Intact mass spectrometry for LCC-ICCG RIP with the top showing the full charge ladder and the bottom showing the deconvoluted spectrum.

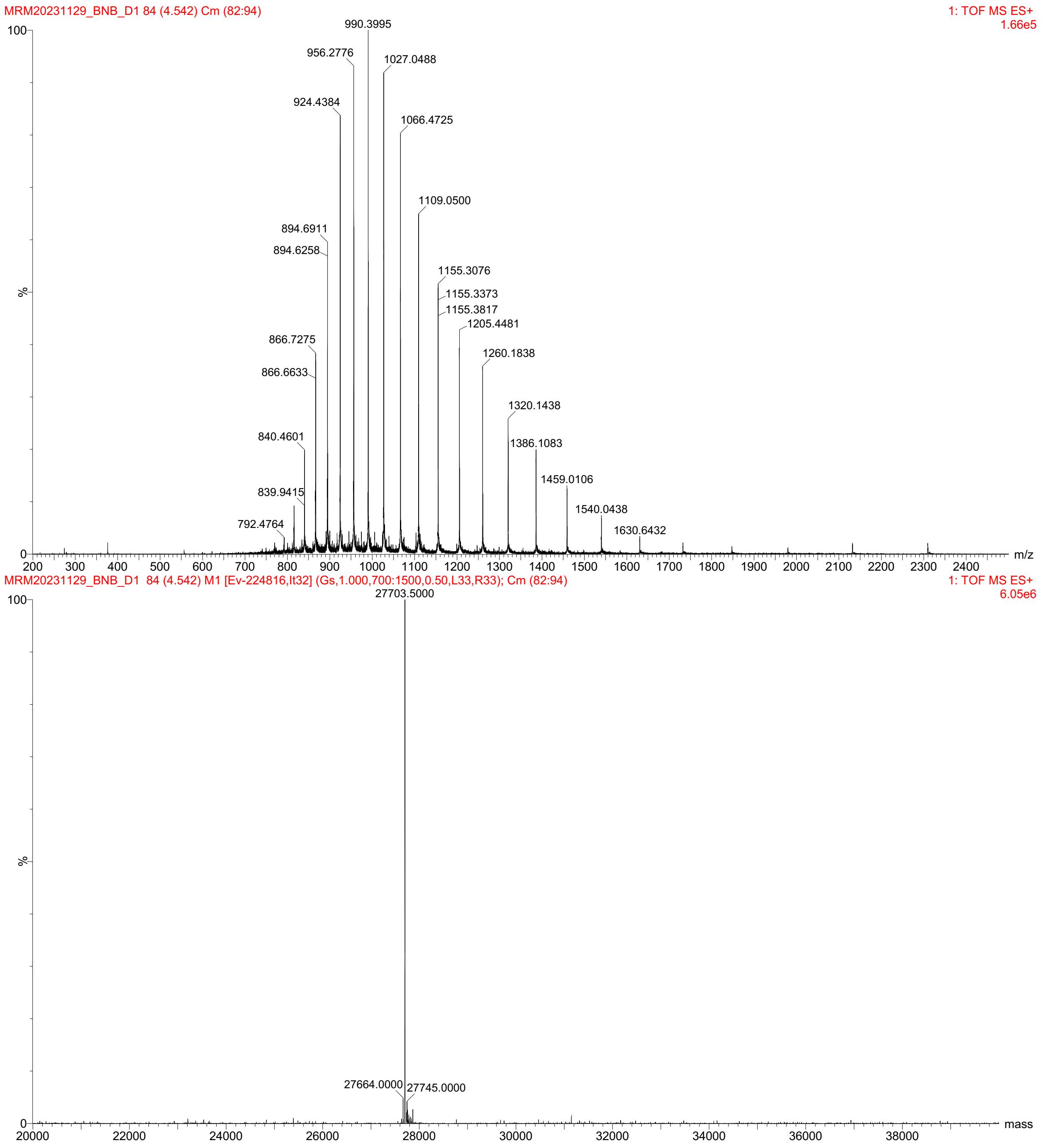


**Supplementary Figure 6.** Intact mass spectrometry for LCC-ICCG DAQI with the top showing the full charge ladder and the bottom showing the deconvoluted spectrum.

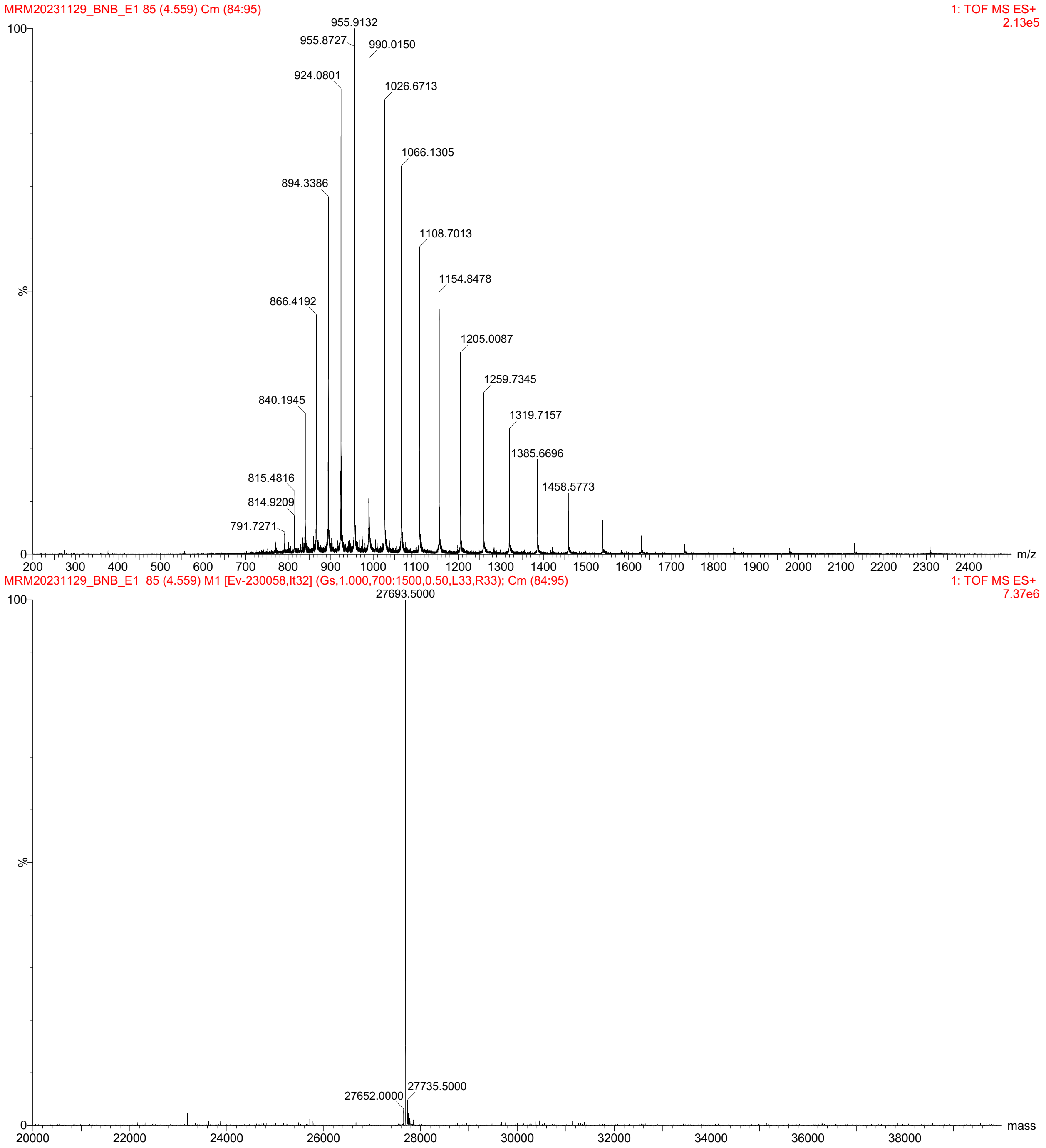


**Supplementary Figure 7.** Intact mass spectrometry for LCC-ICCG I6M with the top showing the full charge ladder and the bottom showing the deconvoluted spectrum.

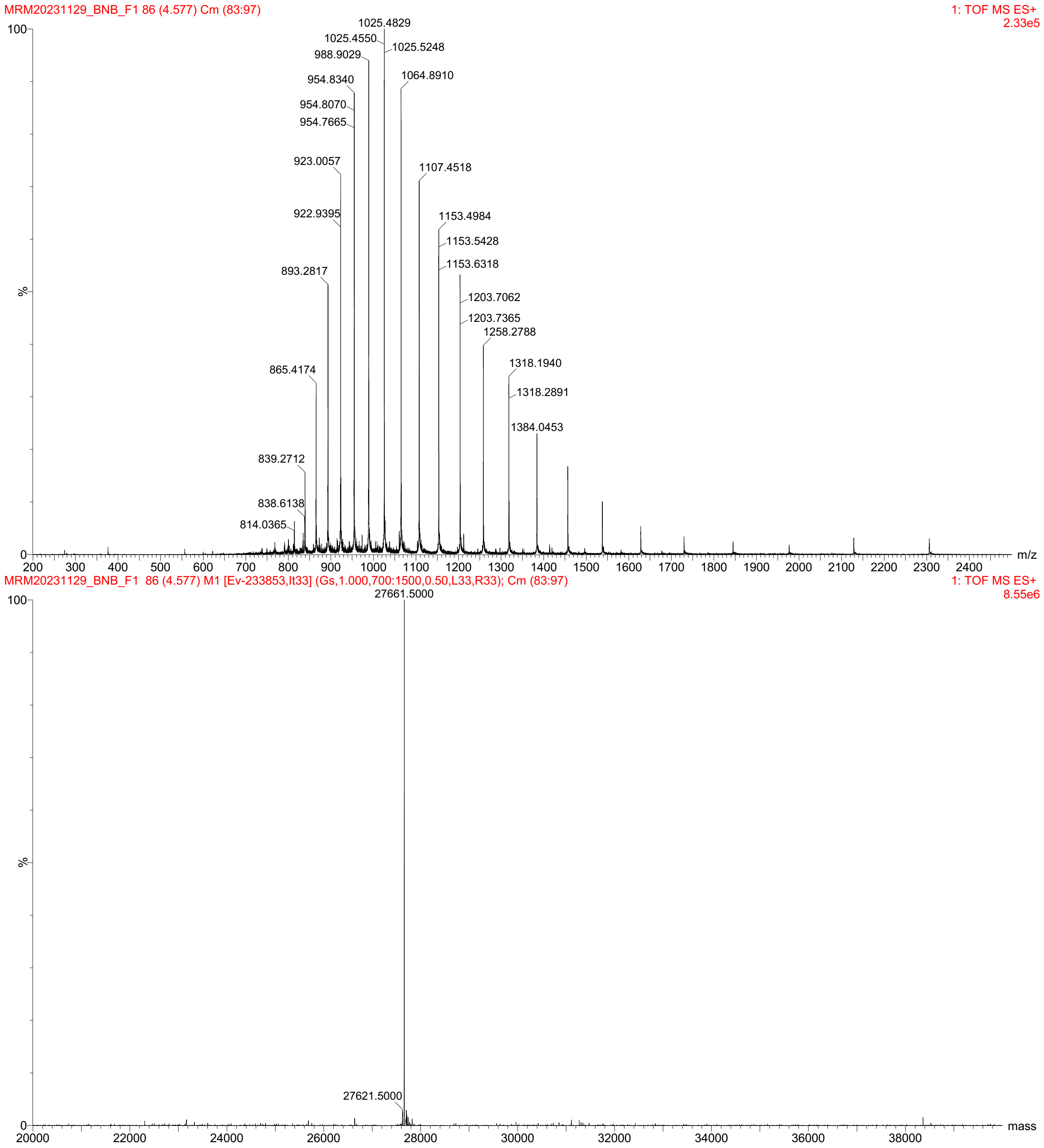


**Supplementary Figure 8.** Intact mass spectrometry for LCC-A2 with the top showing the full charge ladder and the bottom showing the deconvoluted spectrum.

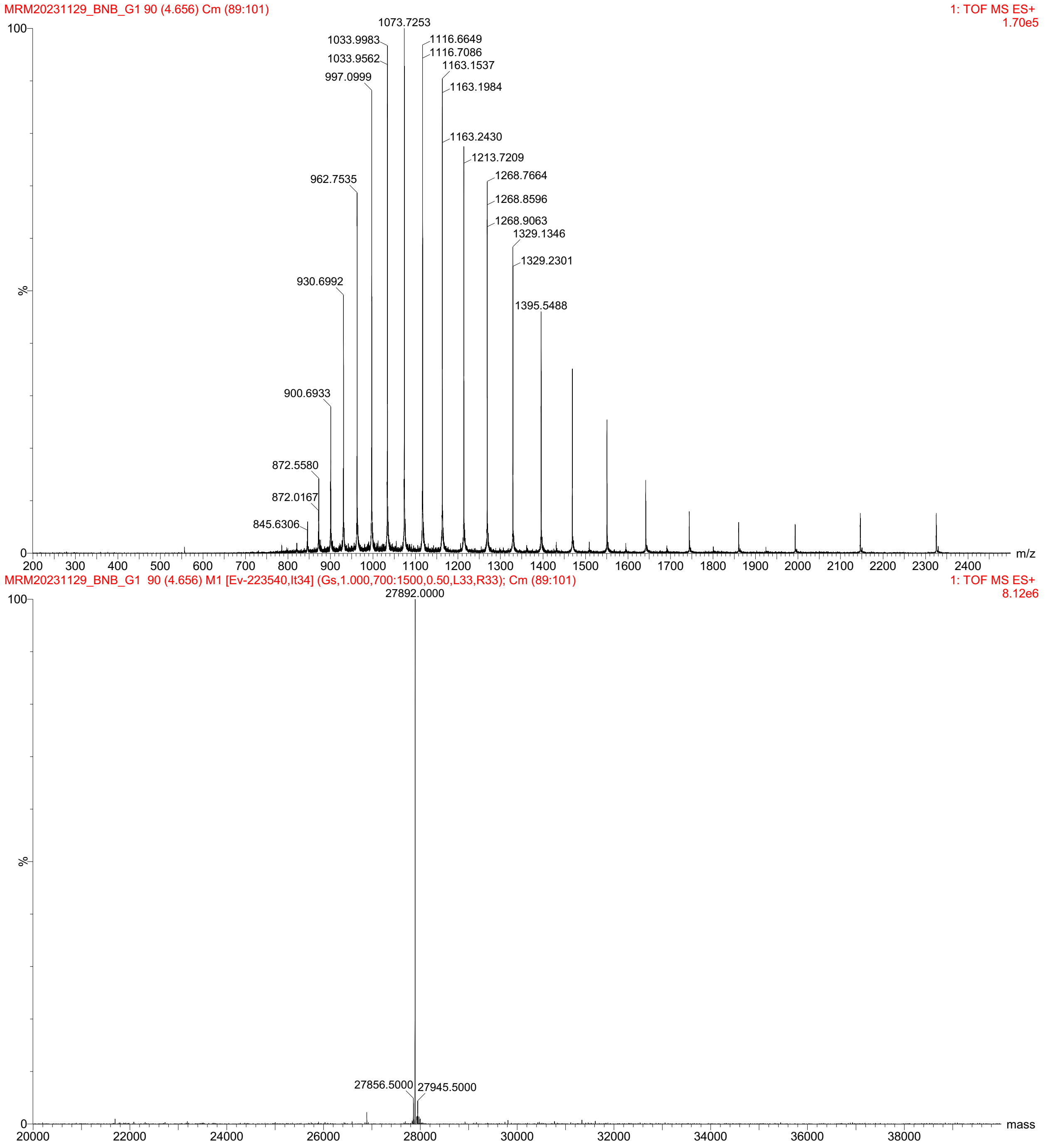


**Supplementary Figure 9.** Intact mass spectrometry for SfCut with the top showing the full charge ladder and the bottom showing the deconvoluted spectrum.

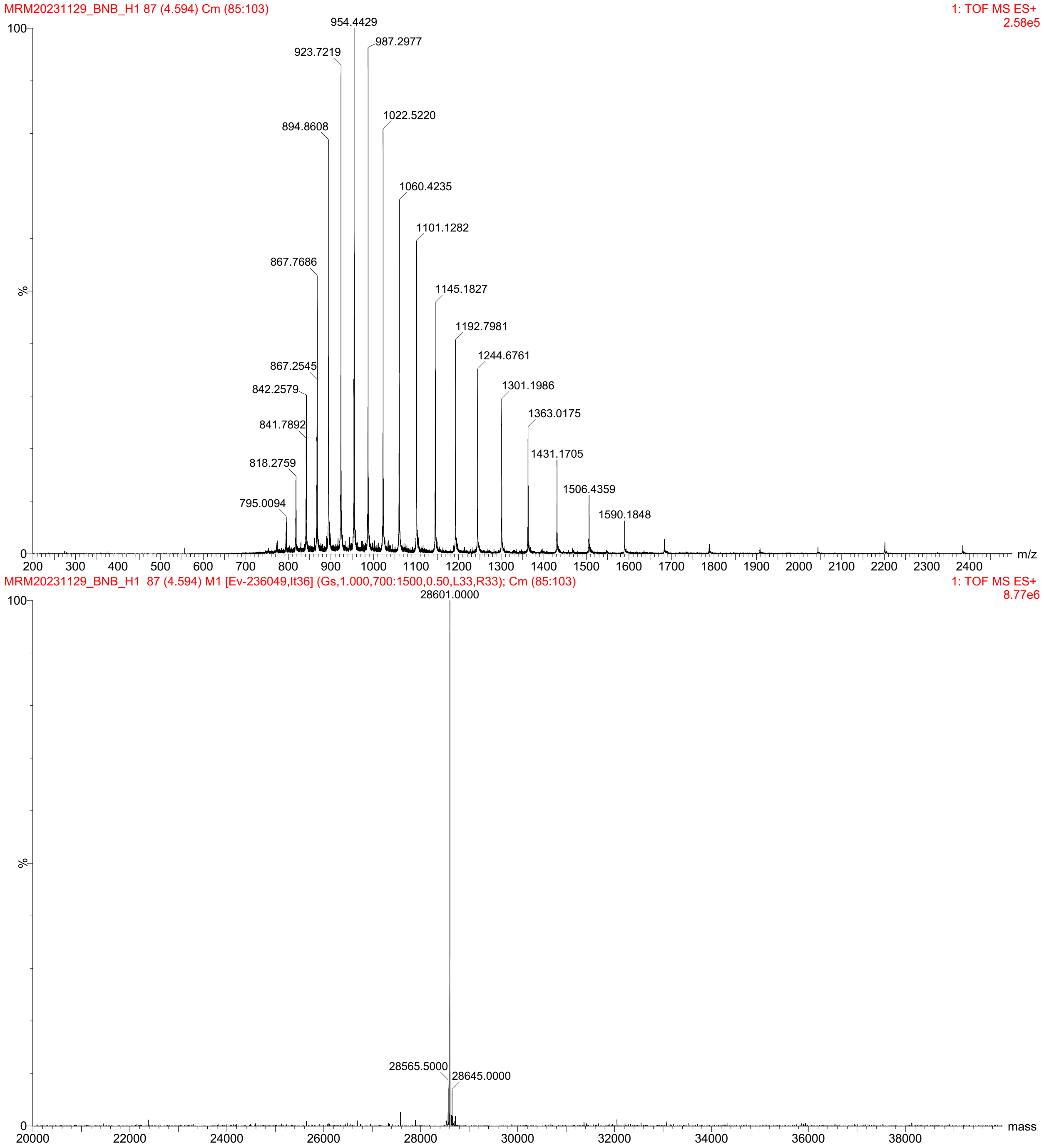


**Supplementary Figure 10.** Intact mass spectrometry for Cut190*SS with the top showing the full charge ladder and the bottom showing the deconvoluted spectrum.

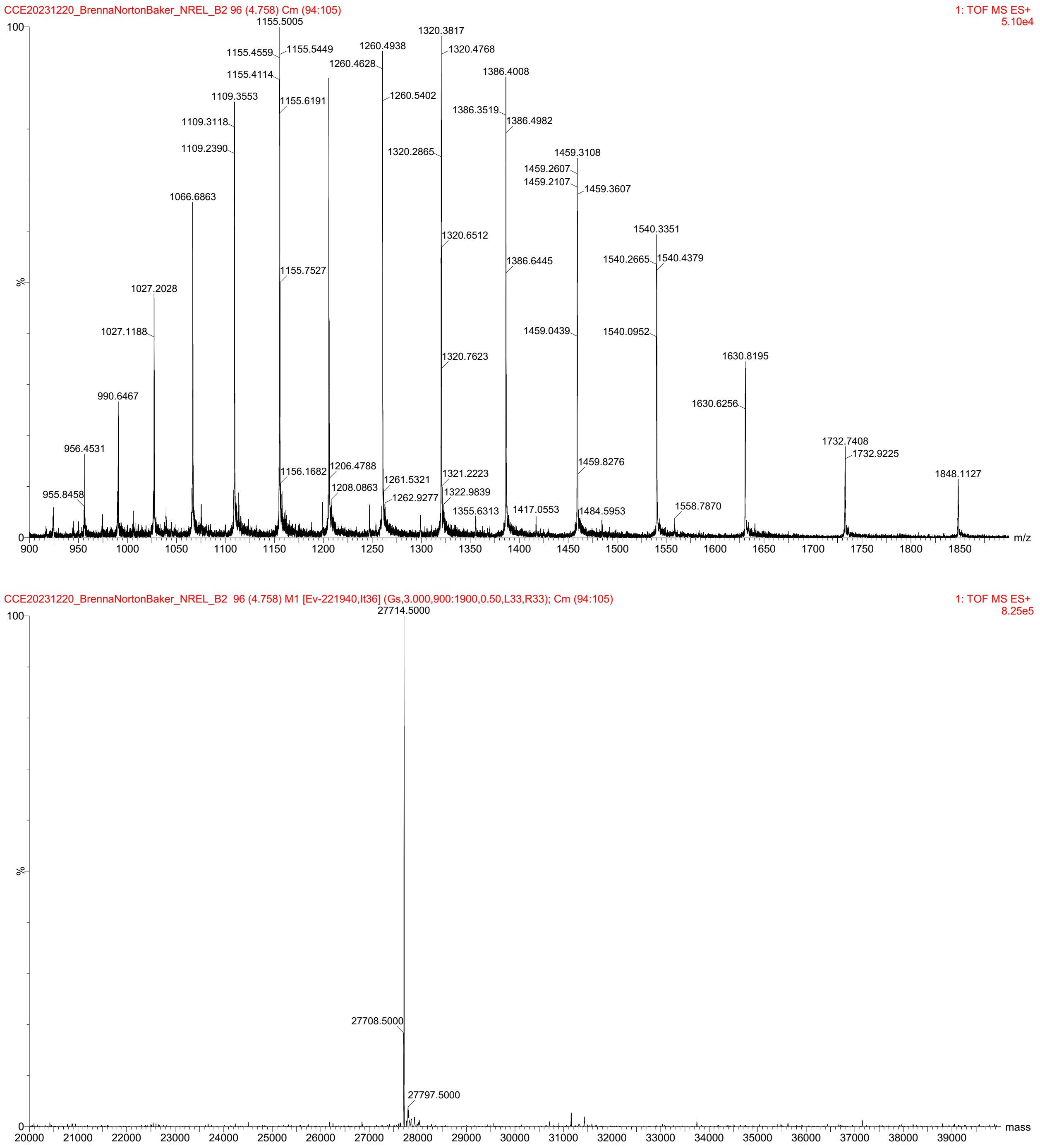


**Supplementary Figure 11.** Intact mass spectrometry for CaPETaseM9 with the top showing the full charge ladder and the bottom showing the deconvoluted spectrum.

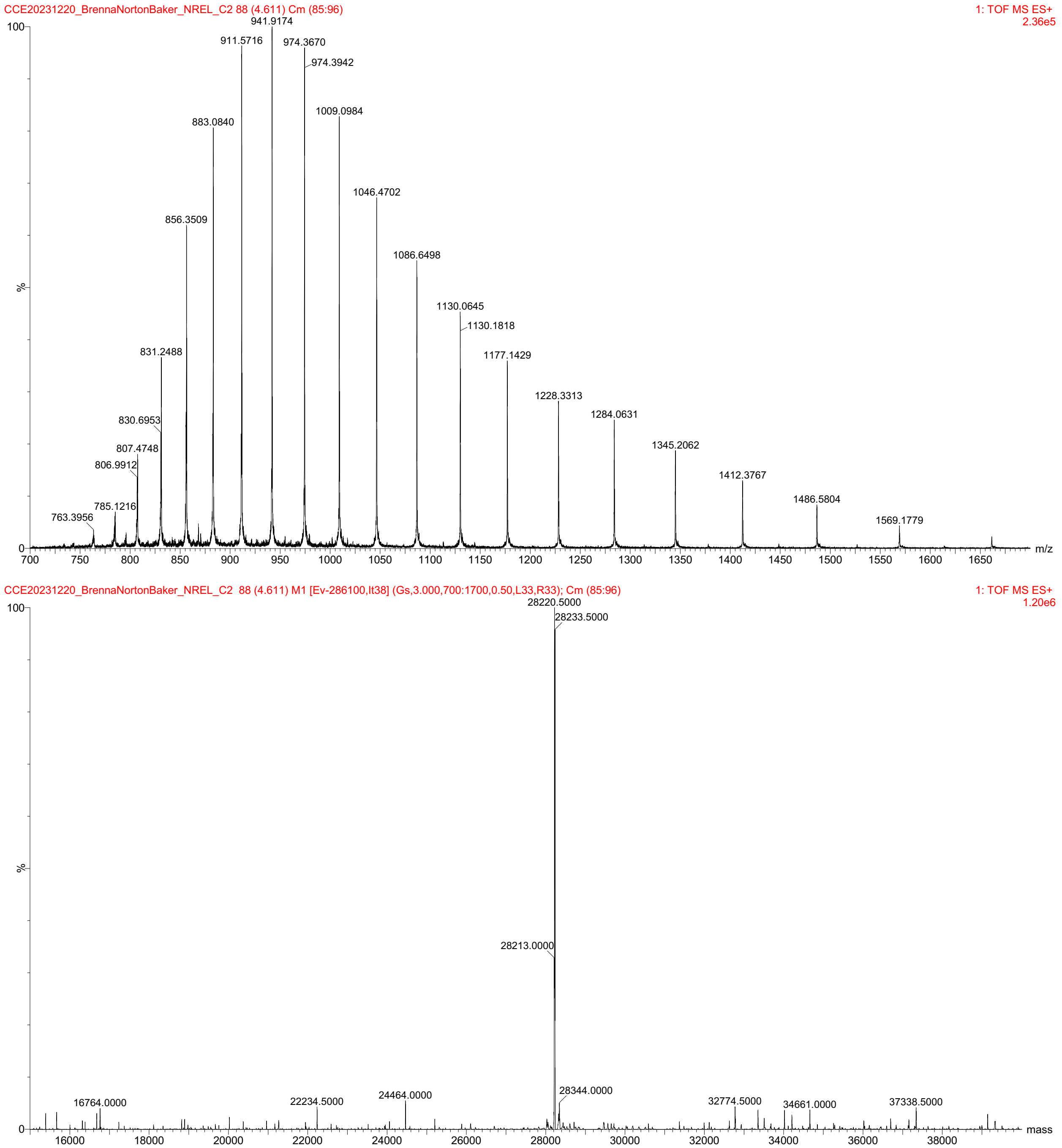


**Supplementary Figure 12.** Intact mass spectrometry for *Tf*Cut2 with the top showing the full charge ladder and the bottom showing the deconvoluted spectrum.

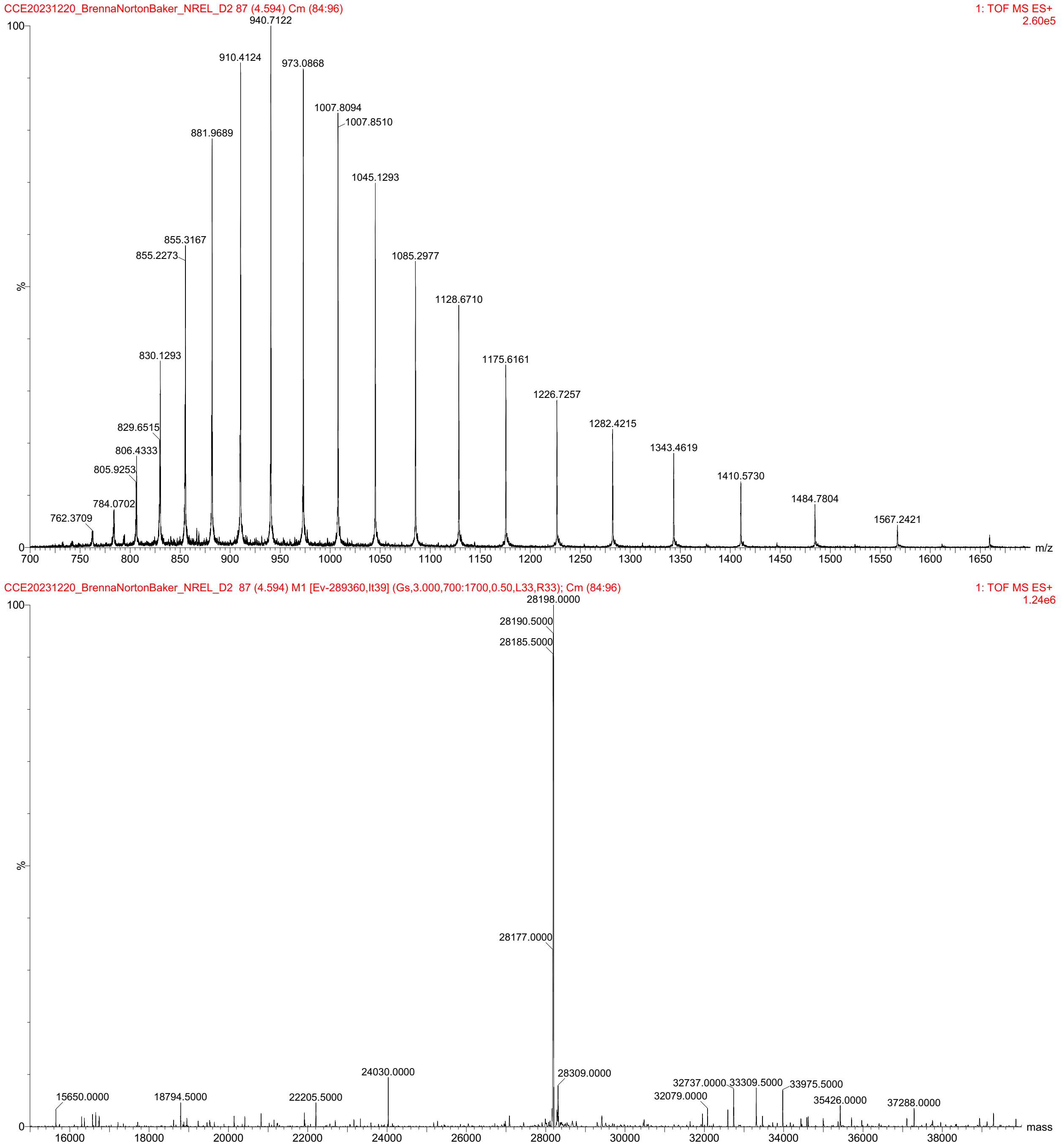


**Supplementary Figure 13.** Intact mass spectrometry for *Tf*Cut2$_{S121P/D174S/D204P}$ with the top showing the full charge ladder and the bottom showing the deconvoluted spectrum.

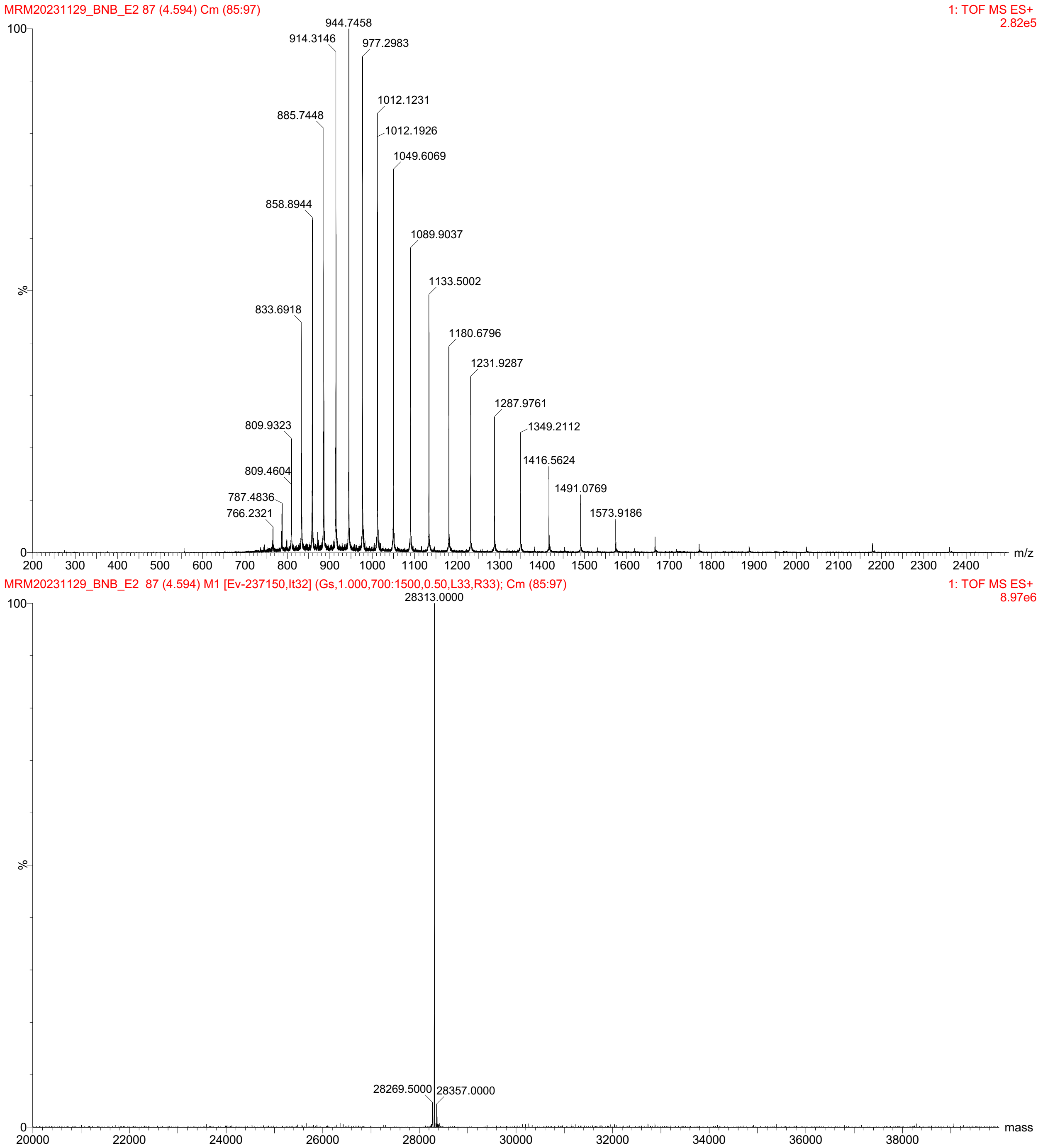


**Supplementary Figure 14.** Intact mass spectrometry for *Tf*Cut2$_{L32E/S113E/T237Q}$ with the top showing the full charge ladder and the bottom showing the deconvoluted spectrum.

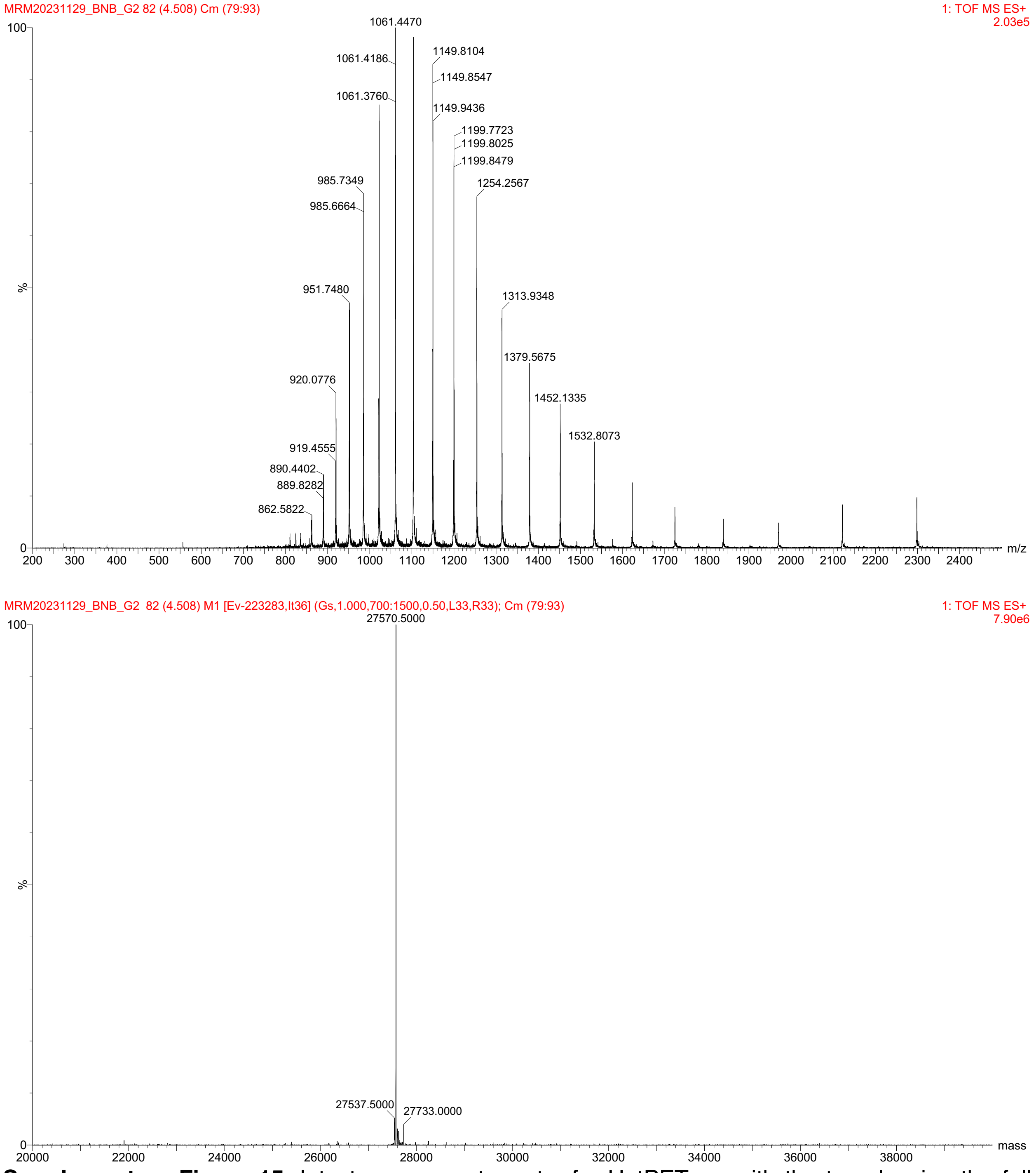


**Supplementary Figure 15.** Intact mass spectrometry for HotPETase with the top showing the full charge ladder and the bottom showing the deconvoluted spectrum.

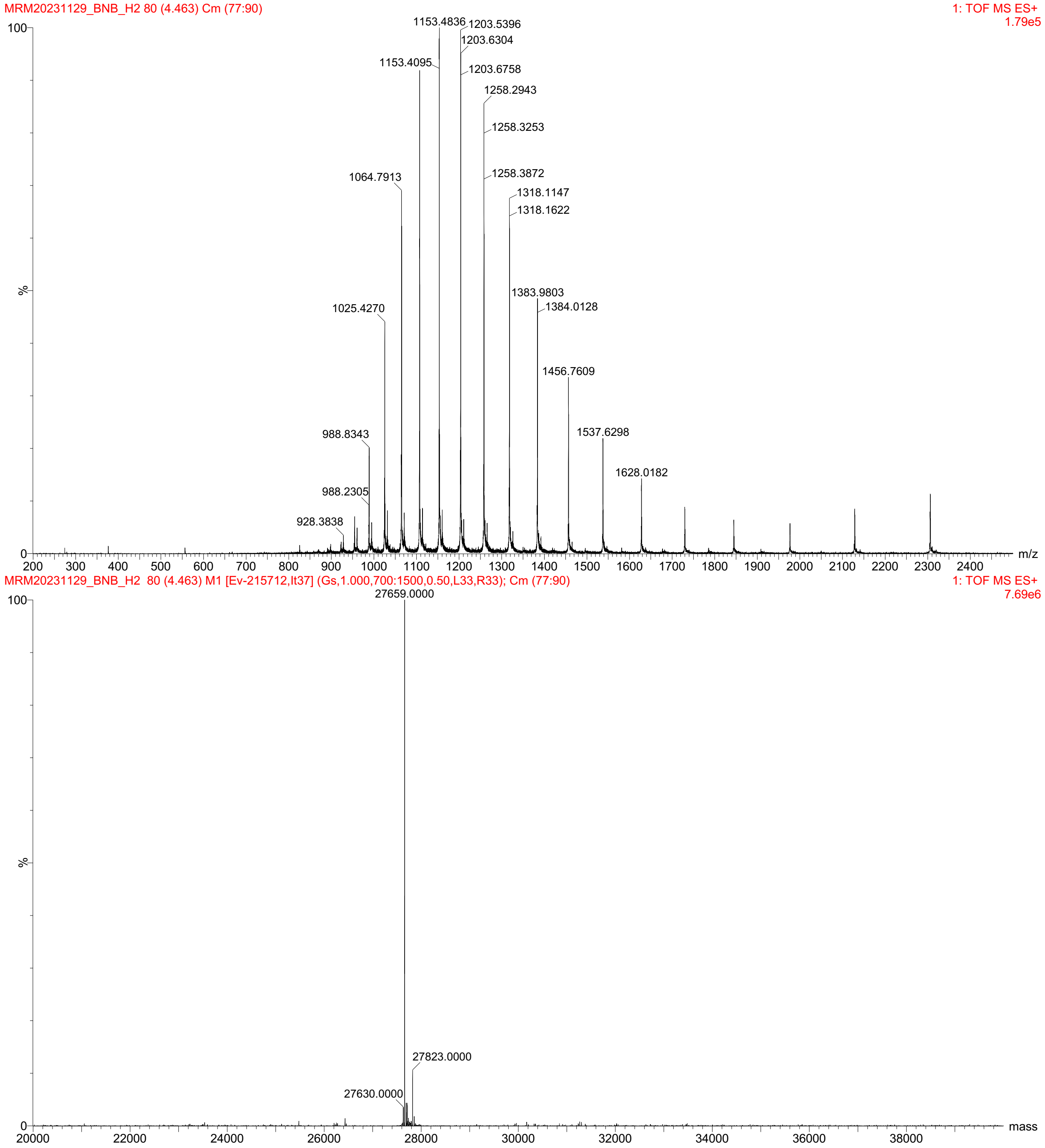


**Supplementary Figure 16.** Intact mass spectrometry for Z1-PETase with the top showing the full charge ladder and the bottom showing the deconvoluted spectrum.

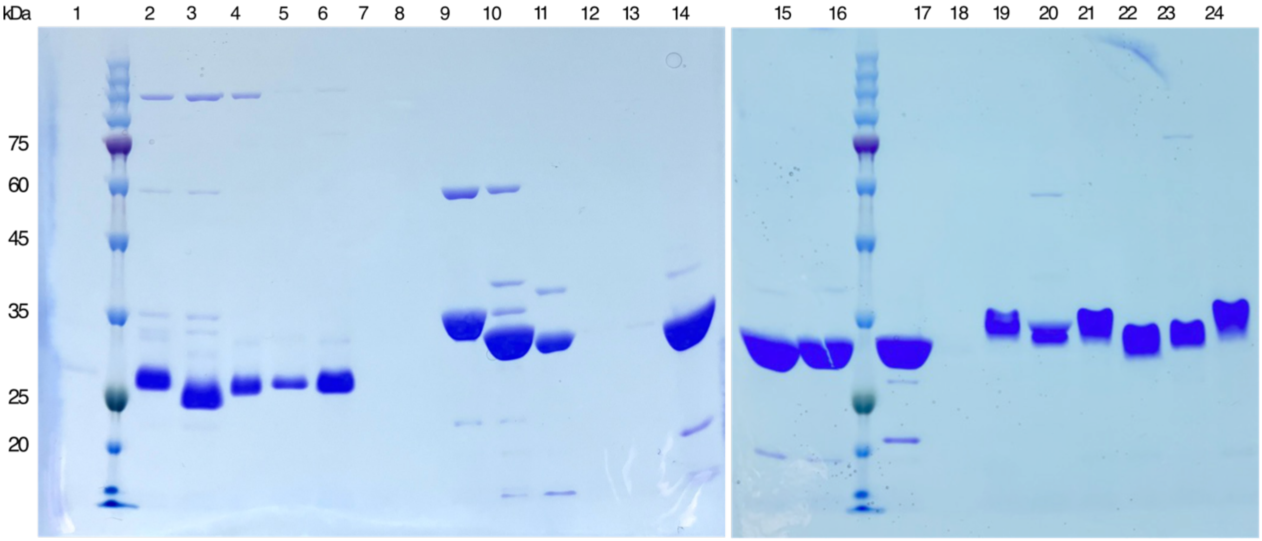


| Number | Name |
|---|---|
| 1 | LCC |
| 2 | LCC-ICCG |
| 3 | LCC-ICCG RIP |
| 4 | LCC-ICCG DAQI |
| 5 | LCC-ICCG I6M |
| 6 | LCC-A2 |
| 7 | BhrPETase |
| 8 | TurboPETase |
| 9 | *Sf*Cut |
| 10 | Cut190*SS |
| 11 | *Ca*PETaseM9 |
| 12 | PES-H1 |
| 13 | PES-H1$_{L92F/Q94Y}$ |
| 14 | *Tf*Cut2 |
| 15 | *Tf*Cut2$_{S121P/D174S/D204P}$ |
| 16 | *Tf*Cut2$_{L32E/S113E/T237Q}$ |
| 17 | RFP |
| 18 | *Is*PETase |
| 19 | ThermoPETase |
| 20 | DuraPETase |
| 21 | FAST-PETase |
| 22 | HotPETase |
| 23 | DepoPETase |
| 24 | Z1-PETase |

**Supplementary Figure 17**. SDS-PAGE post-purification of all enzymes studied.

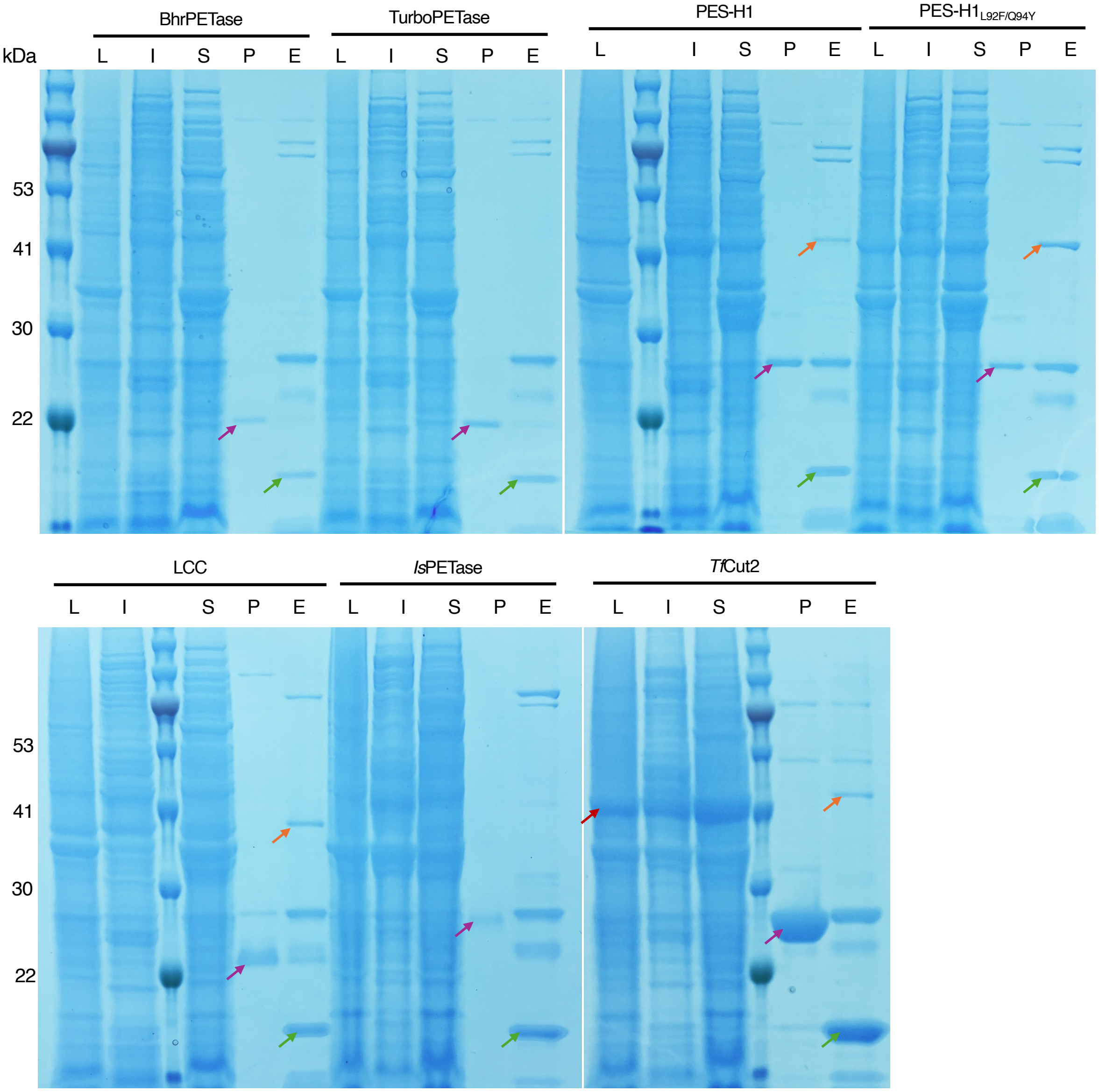


L = whole cell lysate
I = lysate insoluble fraction
S = lysate soluble fraction
P = purified protein, post-cleavage
E = elution from MagBeads, post-cleavage and wash

Overexpression
Purified protein, untagged
Uncleaved tagged-protein
10xHis-SUMO tag

**Supplementary Figure 18**. SDS-PAGE analysis of multiple stages of purification for BhrPETase, TurboPETase, PES-H1, PES-H1$_{L92F/Q94Y}$, LCC, *Is*PETase, and *Tf*Cut2. LCC and *Tf*Cut2 were included as representative examples of a low and high yielding successfully purified protein, respectively. Purified protein (lane P for each sample) was loaded at a higher concentration to enable better visualization compared to **Supplementary Figure 17.** Other then *Tf*Cut2, no clear evidence of an overexpression band (labeled with a red arrow) is seen in the whole cell lysate, suggesting that these proteins were low expressing. A minor amount of purified protein (purple arrows) and SUMO tag (green arrows) is seen for all samples considered ‘unsuccessfully’ purified. Some samples showed evidence of incomplete cleavage (orange arrows) in the imidazole elution of the magbeads post-cleavage.

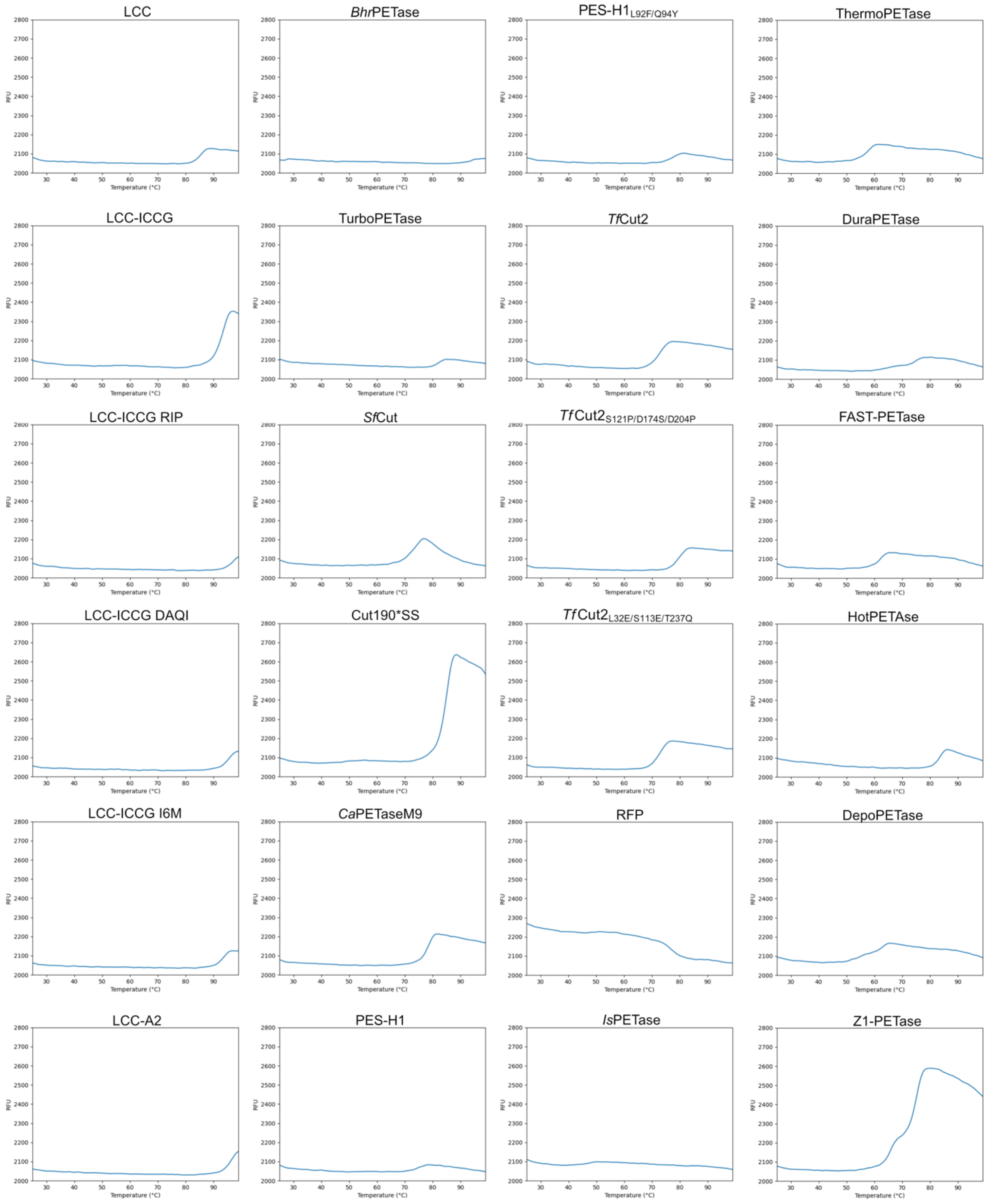


**Supplementary Figure 19.** Differential Scanning Fluorimetry (DSF) data for all enzymes studied. A representative trace for fluorescence as a function of temperature for each enzyme is shown.

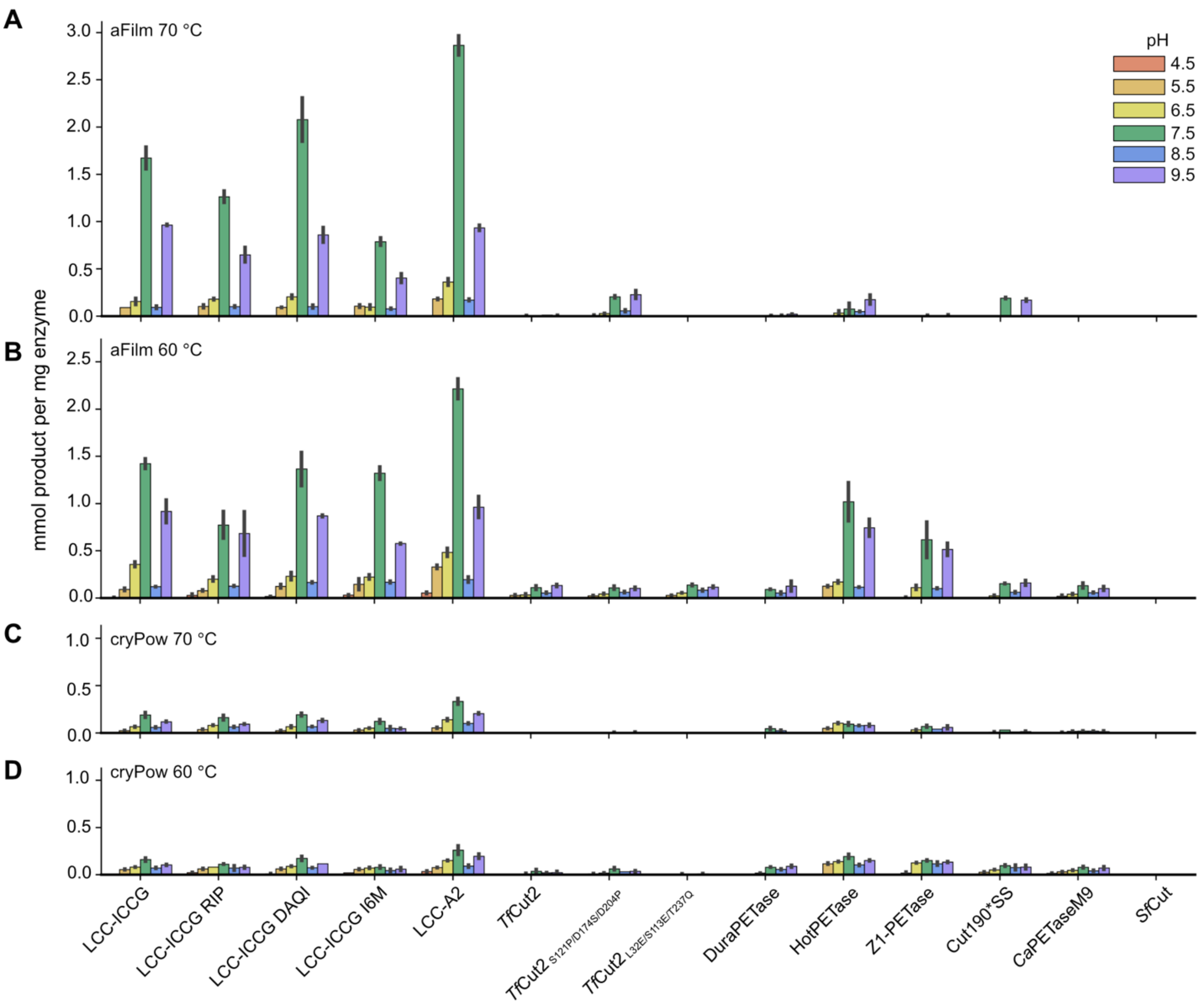


**Supplementary Figure 20.** Enzyme activity data measured at 24 h as determined by UV-Vis analysis for all pH values with A) aFilm at 70 °C, B) aFilm at 60°C, C) cryPow at 70 °C, and D) cryPow at 60 °C. The error bars represent the range between two biological replicates.

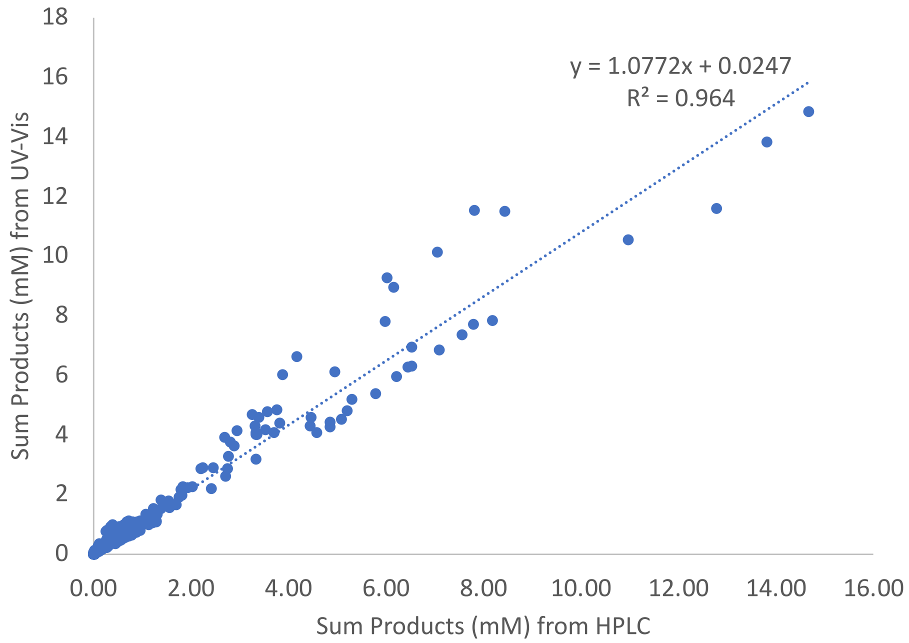


**Supplementary Figure 21.** UV-Vis analysis compared to HPLC analysis of total aromatic product concentration of the PET hydrolysis from all reactions performed for 24 h.

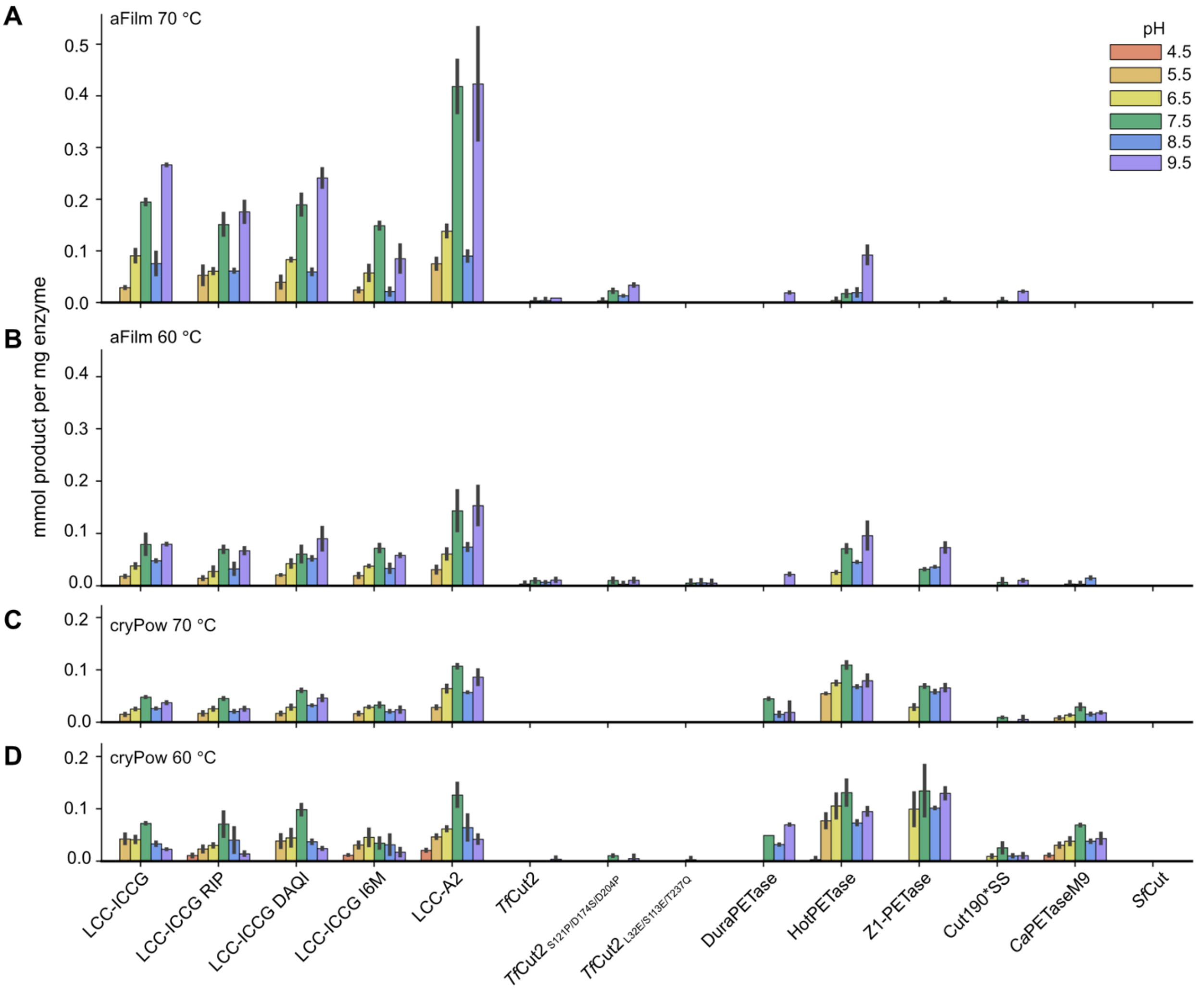


**Supplementary Figure 22.** Enzyme activity data measured at 2 h as determined by UV-Vis analysis for all pH values with A) aFilm at 70 °C, B) aFilm at 60°C, C) cryPow at 70 °C, and D) cryPow at 60 °C. The error bars represent the range between two biological replicates.

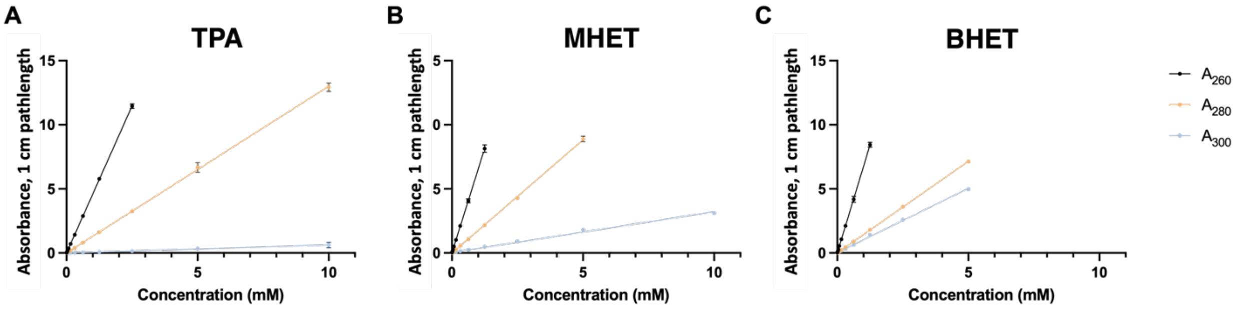


$\varepsilon_{260} = 4.588$ $mM^{-1}\ cm^{-1}$
$\varepsilon_{280} = 1.300$ $mM^{-1}\ cm^{-1}$
$\varepsilon_{300} = 0.06259$ $mM^{-1}\ cm^{-1}$

$\varepsilon_{260} = 6.519$ $mM^{-1}\ cm^{-1}$
$\varepsilon_{280} = 1.767$ $mM^{-1}\ cm^{-1}$
$\varepsilon_{300} = 0.3182$ $mM^{-1}\ cm^{-1}$

$\varepsilon_{260} = 6.3735$ $mM^{-1}\ cm^{-1}$
$\varepsilon_{280} = 1.429$ $mM^{-1}\ cm^{-1}$
$\varepsilon_{300} = 1.001$ $mM^{-1}\ cm^{-1}$

**Supplementary Figure 23.** UV-Vis spectroscopy calibration curves for A) terephthalic acid (TPA), B) mono(2-hydroxyethyl) terephthalate (MHET), and C) bis(2-hydroxyethyl) terephthalate (BHET) to determine molar extinction coefficients at 260, 280 and 300 nm. Data are presented as mean values ± standard deviation (*n*=3).
